# WISH-barcoding of *Salmonella* Typhimurium ATCC14028s strains for population dynamics studies *in vivo*

**DOI:** 10.1371/journal.pone.0349327

**Published:** 2026-10-14

**Authors:** Christopher Schubert, Jeongmin Kim, Nicolas Näpflin, Melanie Hoos, Jemina Huuskonen, Christian von Mering, Wolf-Dietrich Hardt

**Affiliations:** 1 Institute of Microbiology, Department of Biology, ETH Zurich, Zurich, Switzerland; 2 Department of Molecular Life Science and Swiss Institute of Bioinformatics, University of Zurich, Zurich, Switzerland; Islamic Azad University, IRAN, ISLAMIC REPUBLIC OF

## Abstract

**Background:**

Barcoding of isogenic strains is a powerful approach to assess pathogen population dynamics during infection. Here, we adapted WISH-barcoding to *Salmonella* Typhimurium ATCC14028s to evaluate its suitability for pooled infection experiments in streptomycin-pretreated mouse models.

**Results:**

WISH-barcoded wild-type pools showed pronounced population instability, characterized by reproducible strain loss and segregation into high- and low-fitness subpopulations. Whole-genome sequencing identified recurrent mutations in the methyltransferase gene *rsmG* and loss of the P3 plasmid carrying streptomycin resistance in low-fitness strains; neither was observed in Δ*invG* or Δ*ssaV* pools. We propose that *rsmG* mutations were enriched during strain construction carried out under streptomycin selection, following loss of the P3 plasmid. Control experiments demonstrated that *rsmG* mutations and P3 loss are counter-selected *in vivo* and attenuate gut-luminal colonization in streptomycin-pretreated mice.

**Conclusion:**

While population dynamics experiments with ATCC14028s are feasible in principle, wild-type strains are prone to acquiring fitness-altering mutations during *in vitro* construction when using the P3 plasmid and streptomycin, highlighting the need for careful pool validation prior to use and for minimizing antibiotic exposure during strain construction when the strain of interest has no natural resistance, to reduce the risk of selecting mutations.

## Introduction

The gut microbiota confers colonization resistance against invading pathogens through various mechanisms, including nutrient competition (exploitation) and the secretion of antimicrobial compounds and Type VI secretion systems (interference) [1–3]. Despite these barriers, *Salmonella enterica* serovar Typhimurium (*S.* Typhimurium), a Gram-negative enteric pathogen, has evolved strategies to circumvent these defenses, causing foodborne gastroenteritis in humans [4]. As a major cause of diarrheal infections worldwide, *S.* Typhimurium poses a significant public health concern [5]. To investigate the genetic requirements essential for *S.* Typhimurium colonization, genome-wide randomly barcoded transposon sequencing (RB-TnSeq) has previously been employed [6–9]. Transposon mutagenesis works by randomly inserting a transposon into the genome to disrupt genes, allowing identification of genes required for fitness or survival by tracking which insertions are lost under selective conditions [10]. This randomness can be a drawback when focusing on a specific physiological process in hypothesis-driven studies, such as nutrient utilization, particularly when immune responses impose pronounced bottlenecks on the pathogen population — a limitation for approaches relying on large numbers of mutant strains, such as RB-TnSeq [11,12].

To address this limitation, the Wild-Type Isogenic Standardized Hybrid (WISH) barcode, a neutral genetic tag used to track individual strain abundance, was recently established [13] and adapted for *S.* Typhimurium SL1344 [12,14–17]. Here, we aimed to adapt the WISH-barcoding system to ATCC14028s, one of the two most widely used laboratory reference strains for *S*. Typhimurium virulence research. A key requirement is stable colonization, which is essential for robust assessment of mutant fitness in competition experiments. To quantify colonization stability, we calculate the Shannon Evenness Index (SEI), a metric of community evenness that captures how uniformly individual strains are represented within a biological sample, ranging from 0 (strong dominance by a single strain) to 1 (perfectly even distribution across strains) [18,19]. A low SEI reflects the presence of population bottlenecks, such as the granulocyte-dependent bottleneck observed during *S.* Typhimurium infection in the streptomycin-pretreated mouse model between days 2 and 3 post-infection [11]. Similarly, in gnotobiotic mouse models, population bottlenecks occur alongside an increase in the inflammatory response, which is typically observed around day 4 post-infection [12]. Critically, beyond a certain threshold, genuine fitness differences become indistinguishable from stochastic effects imposed by population bottlenecks. Thus, SEI is a key parameter for assessing the robustness of fitness data.

For this reason, we used WISH-barcoded ATCC14028s pools to evaluate colonization stability in the streptomycin-pretreated mouse model. Experiments were performed in two specific pathogen-free (SPF) mouse strains, C57BL/6J and 129S6/SvEvTac. A key distinction between these models is the Nramp1 (Slc11a1) genotype: C57BL/6J mice carry a non-functional allele, rendering them highly susceptible to systemic *S.* Typhimurium infection, whereas 129S6/SvEvTac mice harbor a functional Nramp1 allele that confers increased resistance to systemic dissemination [20]. NRAMP1 is a phagosomal-membrane transporter that effluxes Fe²⁺ and Mn² ⁺ , restricting the availability of these divalent metals to intracellular *Salmonella*; its functional presence in 129S6/SvEvTac confers substantially better control of systemic infection compared with C57BL/6J [21]. In addition to the wild-type strain, we analyzed *invG*- and *ssaV*-deficient ATCC14028s WISH-barcoded pools. InvG is a structural component of the Type III Secretion System 1 (T3SS-1) and is essential for host cell invasion [22–24], whereas SsaV is a core component of T3SS-2, which is required for intracellular survival and systemic dissemination [25,26]. Traversing the tissue barrier constitutes a population bottleneck, the effects of which can extend to the gut-luminal population during reseeding by tissue-lodged *Salmonella*. By comparing Δ*invG* and Δ*ssaV* mutants with the wild-type pool, we assessed whether defects in epithelial invasion or systemic survival alter *S.* Typhimurium population stability in the streptomycin-pretreated mouse model during gut-luminal colonization.

## Results

### Construction of WISH-barcoded ATCC14028s pools to assess colonization stability

Throughout this study, we used the smooth strain ATCC14028s because it retains virulence in mice [27]. To assess the colonization stability of *S.* Typhimurium ATCC14028s in a streptomycin-pretreated mouse model, we rendered the parental strain streptomycin resistant by introducing the P3 plasmid from SL1344 via electroporation prior to WISH-barcoding [28]. We then WISH-barcoded wild-type *S.* Typhimurium ATCC14028s with 10 unique WISH-tags. These tags were coupled to a carbenicillin/ampicillin resistance cassette, enabling quantification of the total tagged population by plating. Individual strain abundances in biological samples were determined by amplicon sequencing [12,13]. In addition to the wild-type parental strain, we created 10 WISH-barcoded strains each in the Δ*invG* and Δ*ssaV* backgrounds (Fig 1A). We used two streptomycin-pretreated mouse models, specific pathogen free C57BL/6J and 129S6/SvEvTac mice. In both models, mice were treated with 25 mg streptomycin prior to infection to reduce the microbiota, allowing *S.* Typhimurium to reproducibly colonize the gut [22]. On the day of infection, mice were inoculated with 5 × 10⁷ CFU by oral gavage. Fecal samples were collected for three consecutive days post-infection. On day three, mice were euthanized and dissected, and in addition to fecal samples, cecal content and systemic organs – including the mesenteric lymph nodes, liver, and spleen – were collected (Fig 1B). To assess the colonization stability of ATCC14028s, all samples, except for organs, were enriched in selective LB (100 µg/ml carbenicillin) at 37°C for 4 hours. Afterwards, genomic DNA (gDNA) was isolated, and the library for amplicon sequencing was prepared through two consecutive PCR reactions (Fig 1C). WISH-barcodes were quantified by amplicon sequencing and analyzed using the mBARq software suite [29], and the resulting counts were used to calculate SEI values (see Methods, S1 Table and S2 Table).

**Fig 1:**
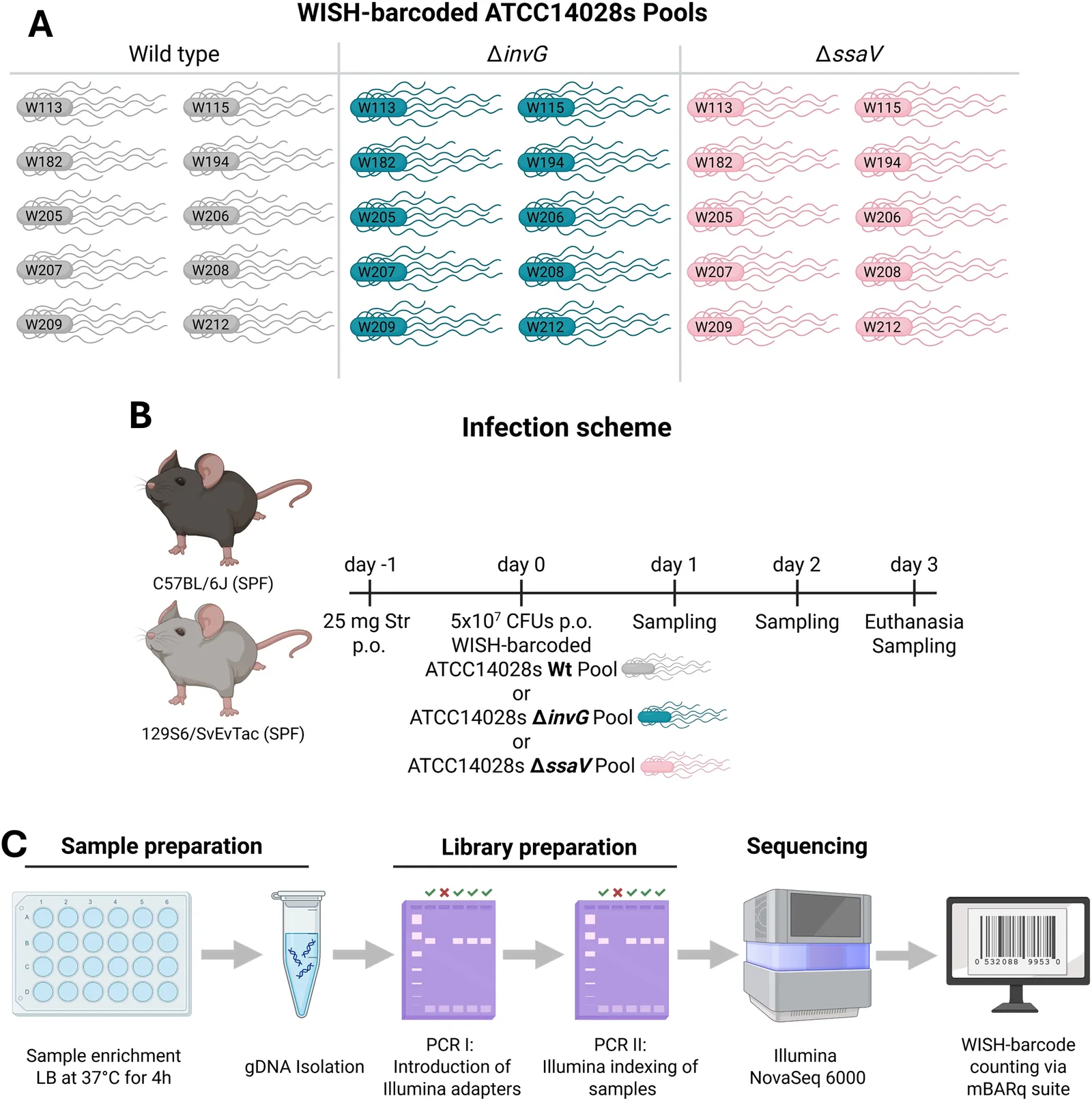
Experimental design of WISH-barcoded ATCC14028s pool infections. (**A**) Composition of WISH-barcoded ATCC14028s pools. The wild-type, Δ*invG*, and Δ*ssaV* pools each consisted of 10 uniquely WISH-barcoded strains. Created in BioRender. Schubert, C. (2026) https://BioRender.com/8twomu6 (**B**) Infection scheme. Streptomycin-pretreated C57BL/6J and 129S6/SvEvTac mice were orally inoculated with 5 × 10^7^ CFU of the indicated WISH-barcoded pools. Fecal samples were collected over three days post-infection, followed by euthanasia and sampling of feces, cecal content, and organs, including liver, spleen, and mesenteric lymph nodes. Created in BioRender. Schubert, C. (2026) https://BioRender.com/8twomu6 (**C**) Library preparation workflow. Samples were enriched in selective LB medium, genomic DNA was isolated, Illumina adapters and sample indices were introduced by PCR, and WISH-barcodes were quantified by amplicon sequencing to determine relative strain abundances. Created in BioRender. Schubert, C. (2026) https://BioRender.com/8twomu6.

### ATCC14028s wild-type populations show high degrees of unevenness when colonizing the gut of streptomycin-pretreated mice

To assess the evenness of gut-luminal ATCC14028s colonization, we compared WISH-barcoded wild-type ATCC14028s pools alongside Δ*invG* and Δ*ssaV* pools (Fig 1A). The evenness of the gut-luminal population was quantified using the Shannon Evenness Index (SEI). A conservative cutoff of 0.9 has been established previously, with samples exhibiting an SEI ≥ 0.9 considered stably colonized [11,12]. In both mouse models, infection with the WISH-barcoded *S.* Typhimurium wild-type pool resulted in a significantly lower SEI compared with the Δ*invG* and Δ*ssaV* pools. In contrast, the SEI of the gut-luminal Δ*invG* and Δ*ssaV* pools remained stable throughout the three days of infection. The Δ*ssaV* pool consistently exhibited significantly higher SEI values in both mouse models than the Δ*invG* pool (Fig 2A, B). Nevertheless, SEI values for both the Δ*invG* and Δ*ssaV* pools remained sufficiently high, indicating stable population dynamics during colonization.

**Fig 2:**
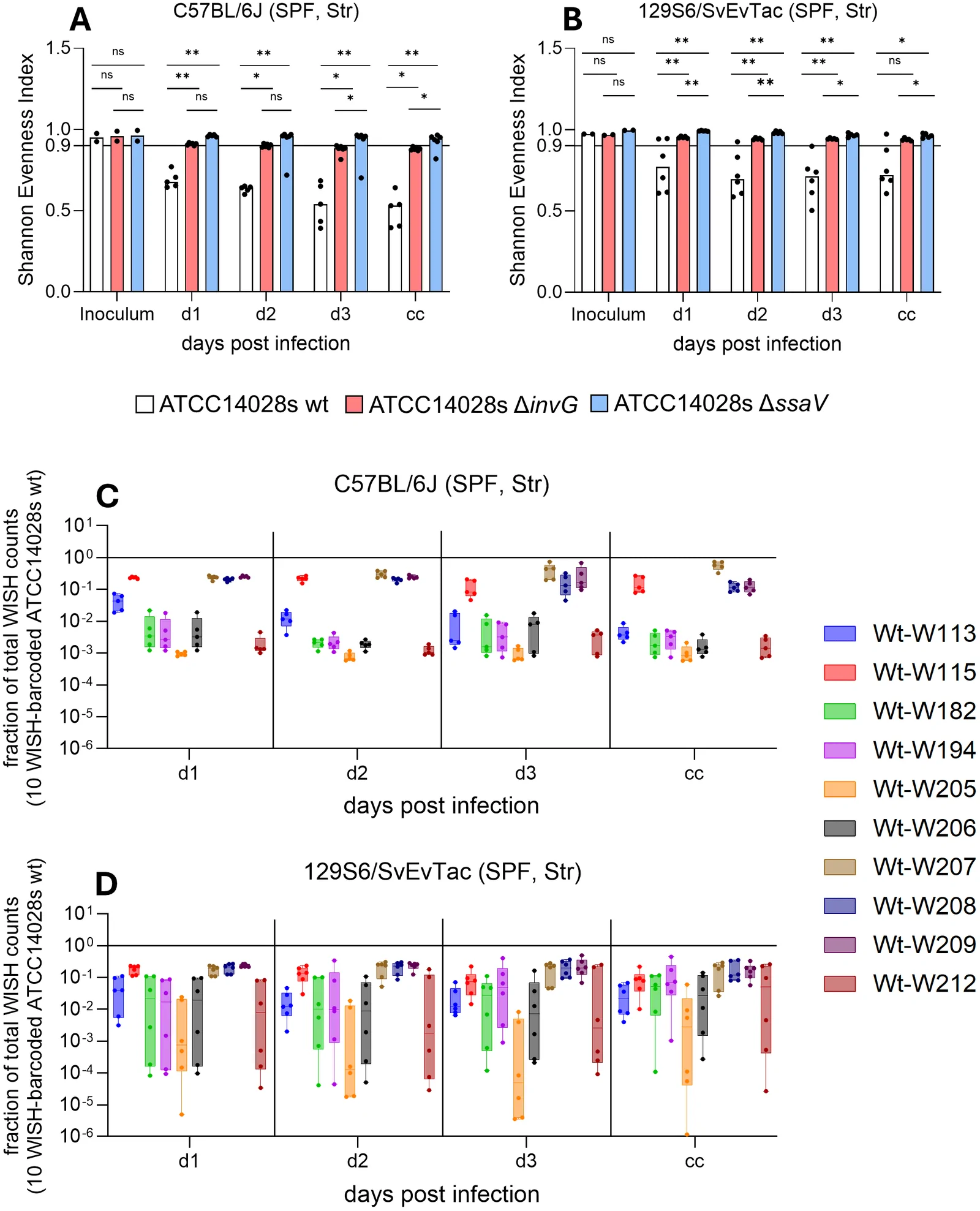
Δ*invG* and Δ*ssaV* mutants have a significantly higher SEI in both mouse models. The Shannon Evenness Index (SEI) was calculated for WISH-barcoded ATCC14028s strains across the three pools: wild type, Δ*invG*, and Δ*ssaV*. SEI values for the streptomycin-pretreated C57BL/6J (**A**) and 129S6/SvEvTac (**B**) models are presented as bar plots, displaying the median and individual data points, at the inoculum (n = 2) and on days 1–3 post-infection and in cecal content (cc; n = 5–6 per group per timepoint). A threshold SEI value of 0.9 is indicated. Panels (**C, D**) depict, for the wild-type pool only, the fraction of individual WISH counts normalized to the total WISH counts across all 10 WISH-barcodes in that pool, in C57BL/6J (**C**) and 129S6/SvEvTac (**D**) mice. Both panels are presented as box-and-whisker plots, showing the median, interquartile range (25th to 75th percentiles), minimum and maximum values, and individual data points. P values were determined using the two-tailed Mann–Whitney U test (ns, not significant; *P < 0.05; **P < 0.01; ***P < 0.001).

To further assess why ATCC14028s wild-type pools yielded lower SEI values, we plotted the relative abundance of each WISH-barcode across all days post-infection, including cecal samples (Fig 2C, D). In the C57BL/6J model, a distinct separation into two clusters was observed: four WISH-barcodes with a high fitness and six with a lower fitness. Notably, all data points are clustered consistently, indicating negligible mouse-to-mouse variability (Fig 2C). If a WISH-barcode had high relative abundance in any individual mouse, it remained dominant throughout the infection period. Interestingly, WISH-barcodes with a high relative abundance in the C57BL/6J model also exhibited a high relative abundance in the 129S6/SvEvTac model, whereas low-abundance WISH-barcodes showed noticeably more pronounced mouse-to-mouse variability (Fig 2D). Importantly, all inocula exhibited SEI values ≥0.95, with each WISH-barcoded strain contributing approximately 9.5% of the total population in both mouse models (S1 Fig A, B). Both experiments were performed independently using the same pre-mixed WISH-barcoded ATCC14028s cryostocks (**see Methods**). This suggests that the competitive advantage of specific WISH-barcoded strains arose before the *in vivo* experiment. We reasoned that this was likely attributable to mutations acquired during the construction of the WISH-barcoded ATCC14028s wild-type strains.

### RsmG partially explains population dynamics in the WISH-barcoded ATCC14028s population

We observed two subpopulations – a low-fitness and a high-fitness group – within the WISH-barcoded wild-type pool (Fig 2C, D), raising the question of whether this difference in colonization resulted from mutations acquired during strain construction *in vitro*. We therefore sequenced all three pools — wild type, Δ*invG*, and Δ*ssaV* — including the parental strains. Interestingly, all WISH-barcoded ATCC14028s strains with attenuated fitness were lacking the P3 plasmid (S2 Fig). It should be noted that streptomycin resistance is not necessarily required to colonize the streptomycin-pretreated mouse model [30,31]. However, we have repeatedly observed that streptomycin resistance confers a significant competitive advantage in co-infection experiments, particularly when one strain lacks resistance in our streptomycin-pretreated mouse models. For this reason, we compared ATCC14028s carrying P3-conferred streptomycin resistance against ATCC14028s lacking P3 in a competition experiment and observed a fitness attenuation in the susceptible strain (S3 Fig). This attenuation was consistent with the fitness defect observed in the pooled experiment. To further investigate this attenuation, we focused on mutations acquired in strains that had undergone P3 plasmid loss. Compared with the parental wild-type strain, four of six WISH-barcoded wild-types that exhibited reduced abundance carried mutations in *rsmG*, which were absent from the WISH-barcoded Δ*invG* and Δ*ssaV* pools (S3-S5 Table). *rsmG* encodes a 16S rRNA methyltransferase that fine-tunes ribosome function and translational fidelity [32]. In Wt-WISH182 and Wt-WISH194, we identified Gly75Val and Gly77Arg substitutions, which replace small, flexible glycine residues with bulky amino acids, likely disrupting protein folding and potentially impairing RsmG function (Fig 3A). In contrast, Wt-WISH206 carried a 13-bp deletion (c.75_87del) in *rsmG*, resulting in a frameshift mutation, whereas Wt-WISH212 contained a mutation introducing an early stop codon (c.469G > T; p.Glu157*). In all cases, these mutations are predicted to disrupt RsmG function. Apart from *rsmG*, Wt-WISH205 instead carried a missense mutation in *rpsL*, the canonical determinant of high-level streptomycin resistance [33].

**Fig 3:**
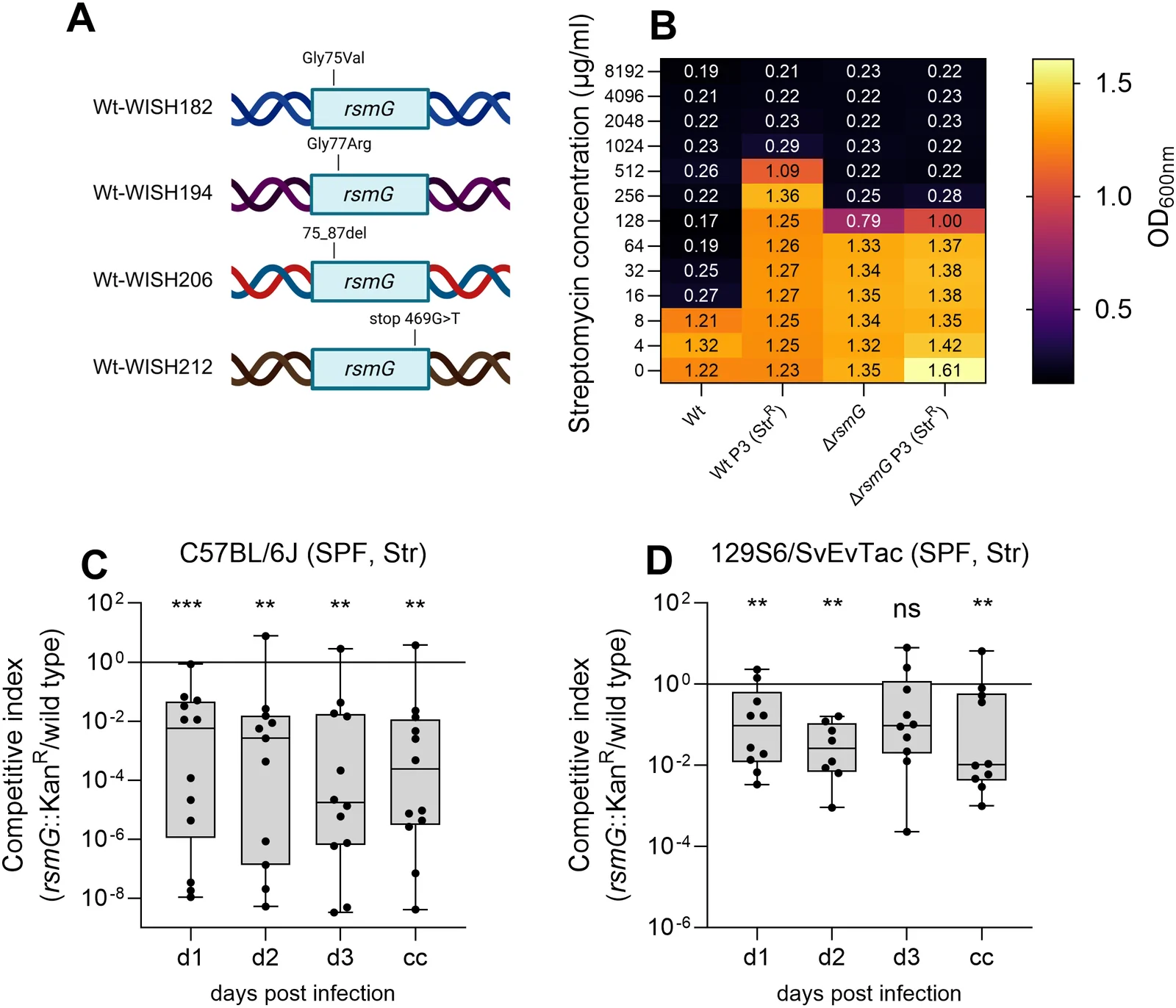
*rsmG* mutants show reduced fitness *in vivo.* (**A**) Schematic overview of mutations identified in *rsmG* in low-abundance WISH-barcoded wild-type strains, including missense substitutions (Gly75Val, Gly77Arg), a 13-bp deletion, and an early stop codon. Created in BioRender. Schubert, C. (2026) https://BioRender.com/8twomu6 (**B**) Minimal inhibitory concentration (MIC) assay for streptomycin showing growth (OD₆₀₀) of wild type, wild type rendered streptomycin resistant with P3 plasmid (Wt Strᴿ), *rsmG* mutant, and *rsmG* mutant with P3 plasmid across increasing streptomycin concentrations. MIC was defined as the lowest concentration at which OD₆₀₀ remained within 0.2 of the no-antibiotic growth-inhibited baseline (i.e., medium-only level), clearly separated from the OD₆₀₀ ≥ ~0.8 seen in wells with visible growth. MIC assays were performed with at least two independent biological replicates, and representative median MICs are reported. (**C, D**) Competitive indices of an *rsmG*::Kan^R^ mutant relative to wild-type ATCC14028s in streptomycin-pretreated C57BL/6J (n = 12) (**c**) and 129S6/SvEvTac (n = 10) (**D**) mice over the course of infection and in cecal content (cc). Each point represents one mouse; boxes indicate median and interquartile range. Statistical significance was assessed using the Wilcoxon matched-pairs signed rank test between wild-type and *rsmG*::Kan^R^ mutant bacterial loads in the respective mouse model (ns, not significant; *P < 0.05; **P < 0.01; ***P < 0.001).

ATCC14028s is not naturally streptomycin resistant; however, *rsmG* mutations have been reported to reduce streptomycin susceptibility [32,34,35]. To test whether this phenotype applied here, we performed minimal inhibitory concentration (MIC) assays with ATCC14028s wild type and the *rsmG* mutant, as well as with both strains rendered streptomycin resistant by introduction of the P3 plasmid from SL1344 [28]. The MIC assay showed that the *rsmG* mutant exhibits a reduction in streptomycin susceptibility compared with the wild type, consistent with low-level resistance, well below the level conferred by P3. The wild type exhibited an MIC of 16 µg/ml, compared with 256 µg/ml for the *rsmG* mutant. In contrast, introduction of the SL1344 P3 plasmid confers high-level streptomycin resistance only in the wild type (MIC of 1024 µg/ml Str). Interestingly, the introduction of the P3 plasmid did not alter the MIC of the *rsmG* mutant (Fig 3B).

### Attenuation of the *rsmG* mutant in the streptomycin-pretreated mouse model

Next, we sought to determine whether *rsmG* mutations could account for the fitness attenuation observed in ATCC14028s in our experiments. Rather than reconstructing each individual mutation, we assessed whether an *rsmG*-deficient strain exhibits a fitness defect during colonization. To this end, we generated an ATCC14028s *rsmG*::Kan^R^ mutant and co-infected streptomycin-pretreated C57BL/6J and 129S6/SvEvTac mice with wild-type ATCC14028s and the *rsmG*::Kan^R^ mutant. Both strains carried the P3 plasmid, so that the comparison was not confounded by the fitness cost of P3 loss. In both mouse models, the *rsmG* mutant was consistently outcompeted by the wild type (Fig 3C, D), in some cases to a degree comparable to that observed in the WISH-barcoded ATCC14028s wild-type pools (Fig 2C, D). These results suggest that the *rsmG* mutations identified in our sequencing data may at least partially explain the reduced abundance of WISH-barcoded strains observed in the initial experiments (Fig 2). Fitness effects of mutations are often condition-dependent, such that a mutation conferring a growth advantage *in vitro* under streptomycin selection can impose a fitness cost during *in vivo* infection. This context-specific trade-off highlights how adaptations beneficial under artificial selection pressures may be detrimental within the complex environment of the mammalian host.

### Reduced fitness of the WISH-barcoded Δ*ssaV* pool at systemic sites

To assess whether deletions of *invG* and *ssaV* affected overall bacterial fitness, we quantified bacterial loads in feces, cecal content, and organs in streptomycin-pretreated C57BL/6J and 129S6/SvEvTac mice. Overall, bacterial loads in the feces and the cecum content were comparable across all parental strains in both models (S4 Fig A, B). In contrast, systemic dissemination differed between strains. In streptomycin-pretreated C57BL/6J mice, the Δ*ssaV* pool exhibited significantly reduced bacterial loads in mesenteric lymph nodes, spleen, and liver compared with the wild-type and Δ*invG* pools, with most liver samples near or below the detection limit (S4 Fig C). A similar trend was observed in the 129S6/SvEvTac model, although the Δ*invG* pool showed lower bacterial loads in spleen and liver compared with C57BL/6J mice (S4 Fig D). To verify inflammatory responses in the gut, we measured the fecal lipocalin-2 (LCN2) levels. In the C57BL/6J model, LCN2 levels were significantly lower in the mice infected with the Δ*invG* and Δ*ssaV* pools compared with the wild type, while in the 129S6/SvEvTac model, only minor, transient differences were observed (S4 Fig E, F). Nevertheless, all strains exceeded the established inflammatory threshold of 500 ng/g feces, previously defined as indicative of colitis [36]. Together, these data indicate that disruption of *invG* or *ssaV* does not substantially impair intestinal colonization, although the Δ*ssaV* pool shows reduced systemic dissemination. The enhanced gut-luminal colonization stability of the Δ*ssaV* and Δ*invG* pools relative to the wild-type pool is most likely attributable to the retention of the P3 plasmid and wild-type *rsmG*, rather than to differences in virulence or inflammatory responses.

## Discussion

We assessed population dynamics of WISH-barcoded ATCC14028s wild-type, Δ*invG*, and Δ*ssaV* pools, each comprising 10 uniquely WISH-barcoded strains (Fig 1A). In streptomycin-pretreated C57BL/6J and 129S6/SvEvTac mouse models, we observed reproducible loss of individual strains within the wild-type ATCC14028s pool (Fig 2A, B). Further analysis revealed a separation into four WISH-barcoded wild-type strains with higher fitness and six with reduced fitness during mouse gut colonization (Fig 2C, D). Whole-genome sequencing revealed that low-fitness WISH-barcoded wild-type strains had lost P3 plasmid-mediated streptomycin resistance and that four of these had additionally acquired mutations in *rsmG*, a gene encoding a 16S rRNA methyltransferase. While *rsmG* mutations partially compensated for P3 plasmid loss by reducing streptomycin susceptibility *in vitro*, they conferred a significant fitness disadvantage during *in vivo* colonization — a cost that, combined with P3 plasmid loss, likely accounts for the overall reduction in competitive fitness observed in these strains (Fig 3C, D and S3 Fig). In contrast, no such population stratification or fitness defects were observed in the Δ*invG* or Δ*ssaV* pools (Fig 2A, S4 Table and S5 Table). Importantly, neither *rsmG* mutations nor P3 plasmid loss were detected in the Δ*invG* or Δ*ssaV* strain pools. Notably, all pools – wild-type, Δ*invG*, and Δ*ssaV* - were constructed using the same workflow and within the same timeframe, arguing against a general methodological artefact. This selective pressure appeared to be specific to the wild-type background, though the underlying reasons for this remain unclear. Notably, wild-type, Δ*invG*, and Δ*ssaV* strains grow comparably in LB during routine inoculum preparation, making growth-rate-dependent differences in mutation accumulation an unlikely explanation for this strain-specific selective pressure. Furthermore, all WISH-barcodes used in this study were previously employed in our studies with *S.* Typhimurium SL1344 [12–15,17,37], in which no fitness effects attributable to specific WISH-barcodes were observed. We therefore consider it unlikely that WISH-barcode identity itself influences *Salmonella* strain fitness in our experimental system.

Streptomycin resistance in *Mycobacterium tuberculosis* is frequently not explained by the classical *rrs* or *rpsL* mutations, prompting the identification of *gidB* (synonymous with *rsmG*) as an additional resistance locus [32]. *gidB* encodes a conserved 16S rRNA m⁷G methyltransferase (modifying G527), and loss-of-function mutations confer low-level streptomycin resistance. Loss-of-function mutations in genes such as *rsmG* are recovered at elevated frequency among clinical isolates, because they tolerate a broad range of mutations without severely compromising viability [32]. Streptomycin exposure, as encountered during *in vitro* strain construction, likely enriches pre-existing *rsmG* variants in ATCC14028s, especially in the context of P3 plasmid loss, thereby providing a mechanistic explanation for the repeated and independent emergence of *rsmG* mutations observed across strains. However, our data demonstrate that both *rsmG* mutations and P3 plasmid loss are counter-selected during infection, as they impose a measurable fitness cost during *in vivo* colonization (Fig 3C, D and S3 Fig). Intriguingly, despite sharing the same experimental workflow, this behavior was observed only in the wild-type background; why the Δ*invG* and Δ*ssaV* strains behaved differently remains an open question. Importantly, these mutations were not present in the parental strain used to generate the 10 uniquely WISH-barcoded derived strains included in the pools (S3-S5 Table). Thus, the mutations were likely acquired during P3-transformation or WISH-barcoding and subsequent *in vitro* culturing, which was partially performed under streptomycin selection.

High-throughput mutant pool approaches enable efficient assessment of colonization fitness but critically depend on stable parental strain behavior [10]. The WISH-barcoding system was recently established [13] and adapted for use in *S.* Typhimurium SL1344 [12,14,15]. Random strain loss has also been reported for SL1344, where it coincides with inflammation-associated bottlenecks in streptomycin-pretreated C57BL/6J mice, typically beginning at day 2 post-infection, and during late-stage infection (days 3–4 post-infection) in the gnotobiotic OligoMM^12^ model [11,12]. Unlike ATCC14028s, SL1344 did not exhibit a tendency to acquire *rsmG* mutations or lose P3 plasmid during *in vitro* manipulation. This may reflect the fact that P3 is the native plasmid of SL1344, potentially conferring greater plasmid stability in this background compared to ATCC14028s, in which P3 was introduced exogenously. The WISH-barcoded ATCC14028s Δ*invG* and Δ*ssaV* pools exhibited more stable colonization dynamics, reflecting the retention of P3 plasmid and the absence of fitness-altering mutations in these strains. The mutation-driven stratification of the wild-type ATCC14028s pool into low- and high-fitness subpopulations is attributable to the inadvertent loss of P3 plasmid and acquisition of *rsmG* mutations during strain construction, both of which imposed a fitness cost during *in vivo* colonization. Given its role as a methyltransferase rather than a direct virulence or colonization factor, RsmG most likely affects fitness indirectly, through altered ribosome function and translational fidelity. Deciphering the precise molecular basis of this fitness cost, however, is beyond the scope of this study.

Collectively, our findings reveal that ATCC14028s can readily undergo *in vitro* mutational changes that were not observed in the Δ*invG* or Δ*ssaV* backgrounds, leading to disrupted population evenness in WISH-barcoded pool experiments *in vivo.* Further work is required to fully understand the basis of this behavior in the ATCC14028s wild-type strain and why it is not observed in the Δ*invG* and Δ*ssaV* backgrounds. Regardless, our findings highlight the importance of validating parental strain stability prior to pooled fitness experiments. This is particularly critical when applying WISH-barcoding approaches that rely on P3-encoded streptomycin resistance in ATCC14028s to assess fitness in streptomycin-pretreated mice.

## Methods

### Ethics statement

All animal experiments were reviewed and approved by the Kantonales Veterinäramt Zürich and performed in accordance with Swiss federal legislation (Tierschutzgesetz SR 455, Tierschutzverordnung SR 455.1) under licences ZH158/19, ZH108/22 and ZH109/2022, and in line with the recommendations of the Federation of European Laboratory Animal Science Associations (FELASA). All procedures described here were classified as severity grade 2 (moderate) under the Swiss severity classification, and no procedure exceeded this grade. Reporting follows the ARRIVE 2.0 guidelines [39].

### Anaesthesia, analgesia and measures to alleviate suffering

No surgical or otherwise invasive interventions were performed in this study, and consequently no anaesthesia or analgesia was administered. All procedures were non-surgical and of very short duration: streptomycin pretreatment and bacterial inoculation were carried out by oral gavage, taking a few seconds per animal and performed by personnel formally trained and certified for this procedure (FELASA/LTK Module 1), and fecal pellets were collected non-invasively from freely moving mice. The deliberate omission of analgesia and anaesthesia is explicitly foreseen and justified in the approved licences (ZH108/22, ZH109/2022) on scientific grounds: opioid and non-opioid analgesics substantially alter gut microbiota composition, non-steroidal anti-inflammatory drugs suppress prostaglandin-dependent intestinal inflammatory signalling. Administration of such compounds would therefore have directly confounded the microbiota-dependent colonization and inflammation read-outs of this study, while providing no welfare benefit for procedures that cause only brief, minimal handling stress [40,41].

Several measures were taken to minimise distress. Mice were acclimatized to the experimental holding conditions for at least seven days before the start of any procedure and were inspected for general well-being prior to enrolment. Animals were group-housed in individually ventilated cages with nesting material and environmental enrichment, with food and water available ad libitum. All experiments were terminated at a fixed endpoint of day 3 post-infection, deliberately chosen to precede the onset of severe systemic salmonellosis, which develops from day 5–6 post-infection in susceptible C57BL/6J mice; this schedule caps the maximum burden experienced by any animal. Animals were monitored daily using a standardized score sheet (behaviour, coat condition, posture, stool consistency, weight loss or body condition score, and skin turgor; see “Humane endpoints”), and were additionally inspected 10 and 30 min after gavage for signs of aspiration, with immediate euthanasia in case of respiratory distress. In accordance with the 3R principles, the WISH-barcoding approach used here allowed ten isogenic strains to be tracked simultaneously within a single animal, reducing the number of mice required by approximately ten-fold relative to single-strain infections. At the end of the experiment, and at any humane endpoint, mice were euthanised by CO_2_ asphyxiation using a rising-concentration protocol in the home cage; death was verified by the absence of respiration, heartbeat and reflexes, and was subsequently confirmed by opening of the abdominal and thoracic cavities during organ sampling. No animal reached a humane endpoint and no unscheduled deaths occurred during any experiment.

### Humane endpoints

All animals were monitored daily throughout the experiment. Humane endpoint scoring assessed six categories: behavior, coat condition, posture, stool consistency, weight loss or body condition score, and skin turgor. Each category was scored 0 (normal), 2 (moderate), or 3 (severe). Animals were immediately euthanized if they scored 3 in any single category, or if a sum score of ≥4 was sustained over 12 hours. Animals with a sum score of ≥2 were re-evaluated within 10–12 hours. Additionally, mice were inspected 10 and 30 minutes after orogastric gavage for signs of aspiration. All experiments were terminated at a fixed endpoint of day 3 post-infection by CO_2_ asphyxiation. No animals met the criteria for humane endpoints during any experiment. No deaths occurred prior to the scheduled experimental endpoint.

### Animals

Male and female mice aged 8–12 weeks were used and randomly assigned to experimental groups. The consideration of animal sex was not included in the study design. All mice were maintained on the normal mouse chow (Kliba Nafag, 3537; autoclaved; per weight: 4.5% fat, 18.5% protein, 50% carbohydrates, 4.5% fiber). The mice originated from C57BL/6J or 129S6/SvEvTac breeders initially obtained from Jackson Laboratories. Mice with a normal complex microbiota were specific pathogen-free (SPF) and bred under full barrier conditions in individually ventilated cage systems at the EPIC mouse facility of ETH Zurich (light/dark cycle 12:12 h, room temperature 21 ± 1 °C, humidity 50 ± 10%). All studies were conducted in compliance with ethical and legal requirements and were reviewed and approved by the Kantonales Veterinäramt Zürich under licenses ZH158/19, ZH108/22 and ZH109/2022.

### Bacteria and culture conditions

All *Salmonella* strains used in this study were derived from the smooth variant *Salmonella* Typhimurium ATCC14028s [27]. Prior to WISH-barcoding, the parental ATCC14028s strain was rendered streptomycin resistant by electroporation with the mobilizable plasmid P3 (accession HE654726.1) from *S.* Typhimurium SL1344 [28]; all wild-type, Δ*invG*, and Δ*ssaV* parental strains used to construct the WISH-barcoded pools carry this plasmid. Strains are listed in S6 Table. All strains were routinely grown overnight at 37 °C in Lysogeny broth (LB) with agitation, supplemented with the appropriate antibiotics where required (carbenicillin 100 µg/ml, kanamycin 50 µg/ml, streptomycin 50 µg/ml). Strains were stored at −80°C in peptone glycerol broth (2% w/v peptone, 5% v/v glycerol (99.7%)). Custom oligonucleotides were synthesized by Microsynth and are listed in S7 Table; plasmids used in this study are listed in S8 Table.

### Homologous recombination by Lambda Red

*rsmG* knockout strains were generated using the lambda-red single-step recombination protocol [42]. Primers were designed with ~40 bp homology arms targeting the genomic region of interest and a 20 bp priming region corresponding to the antibiotic resistance cassette. PCR amplification was performed using plasmid pKD13 as the template for kanamycin resistance (S8 Table). DreamTaq Master Mix (Thermo Fisher Scientific) was used according to the manufacturer’s instructions. PCR products were purified using the NucleoSpin DNA purification kit (Macherey-Nagel). SL1344 harboring the pKD46 plasmid was cultured for 3 h at 30 °C to early exponential phase, followed by induction with L-arabinose (10 mM; Sigma-Aldrich) for 20 min. Cells were washed with ice-cold 10% (v/v) glycerol and concentrated 100-fold. Purified PCR products were introduced by electroporation (1.8 kV, 5 ms), followed by recovery in SOC medium (SOB premix; Roth GmbH, supplemented with 50 mM D-glucose) for 2 h at 37 °C, and plating on selective LB agar. Successful gene knockouts were confirmed by PCR and agarose gel electrophoresis, followed by Sanger sequencing (Microsynth AG). Antibiotic resistance cassettes were subsequently excised using FLP recombinase-mediated recombination [43].

### Homologous recombination by P22 phage transduction

The WISH-barcodes were introduced into ATCC14028s via P22 phage transduction from previously published SL1344 strains [12] (S6 Table). P22 phages carrying the respective WISH-barcodes were incubated with the recipient ATCC14028s strains (wild type, Δ*invG*, or Δ*ssaV* parental strains) for 15 min, followed by plating on selective LB agar plates. To ensure the removal of residual phages, transduced colonies were streaked twice consecutively on selective LB-agar plates overnight. To confirm the absence of P22 phage contamination, colonies were screened using Evans Blue Uranine (EBU) LB-agar plates (0.4% w/v glucose, 0.001% w/v Evans Blue, 0.002% w/v Uranine). WISH-tags were integrated at a fitness-neutral locus between the pseudogenes *malX* and *malY*, as previously described [44]. It is important to note that all parental ATCC14028s strains – wild type, Δ*invG*, and Δ*ssaV* - were rendered streptomycin resistant via transformation with the P3 plasmid prior to the introduction of the WISH-barcodes.

### Preparation of the ATCC14028s wild type, Δ*invG*, or Δ*ssaV* pools

Each strain from the WISH-barcoded ATCC14028s wild type, Δ*invG*, or Δ*ssaV* pools was inoculated in selective LB medium containing carbenicillin (100 µg/ml) and grown overnight at 37°C to maintain selection for the WISH-barcode. The overnight culture was then diluted 5% (v/v) into fresh LB medium without antibiotics and grown at 37°C for 4 h until reaching late exponential phase. At this stage, strains were pooled in equal volumes, centrifuged (4,500 × g, 4 °C, 15 min), and the resulting cell pellets were resuspended in peptone-glycerol medium to 10% (v/v) of the original volume and aliquoted into 100 µl cryotubes for storage at −80 °C. This approach enables rapid use of WISH-barcoded ATCC14028s pools, as 20 µl of the pooled stock can be subcultured in LB medium for only 4 h prior to infection.

### Animal experiments

Mice were pretreated with 25 mg streptomycin in 50 µl sterile water by oral gavage 24 h prior to infection [22]. For the ATCC14028s pool experiments, 8- to 12-week-old mice were infected by oral gavage with 5 × 10^7^ CFU in 50 µl. Inocula were prepared from a 4 h subculture (LB medium, from pooled cryostocks), washed once in PBS, and normalized to OD₆₀₀ = 2. Mice were euthanized by CO_2_ asphyxiation on day 3 post-infection. Fresh fecal pellets and whole cecal content were weighed prior to homogenization in 500 µl PBS with a metal bead using a TissueLyser (Qiagen); mesenteric lymph nodes, spleen, and liver were collected at euthanasia, weighed, and homogenized in the same manner. Homogenates were then briefly centrifuged (200 × g, room temperature, 2 min) to sediment debris. Serial ten-fold dilutions of the supernatant were prepared in 96-well microtiter plates and plated on MacConkey agar; colonies were counted the next day from plates yielding 10–300 colonies. CFU per gram of feces, cecal content, or organ was calculated as:


$$\begin{array}{c} \text{CFU}/\text{g feces}=\frac{\text{CFU}\times \text{dilution factor}\times \text{resuspension volume }(\text{ml})}{\text{volume plated }(\text{ml})\times \text{fecal mass }(\text{g})} \end{array}$$

Samples with no colonies at the lowest dilution tested were assigned the assay’s limit of detection.

### Sample preparation for amplicon sequencing and WISH-barcode quantification

After homogenization, fecal and cecal samples (125 µl) were enriched in 1 ml selective LB medium containing 100 µg/mlcarbenicillin for 4 h at 37 °C to select for WISH-barcoded strains. Bacterial cells were then pelleted, the supernatant was discarded, and the pellets were stored at −20 °C. DNA was extracted from thawed pellets using a commercial kit (Qiagen DNeasy Mini Kit) according to the manufacturer’s instructions. For PCR amplification of WISH-barcodes, 2 µl of genomic DNA and 0.5 µM of each primer (WISH_Illumina_fwd and WISH_Illumina_rev; see S7 Table) were used with DreamTaq Master Mix (Thermo Fisher Scientific). The reaction was conducted with the following cycling program: initial denaturation step at (1) 95°C for 3 min followed by (2) 95°C for 30 sec, (3) 55°C for 30 sec, (4) 72°C for 20 sec for (5) 25 cycles, and a terminal extension step at (6) 72°C for 10 min. PCR products were column-purified using the NucleoSpin Gel and PCR Clean-up kit (Macherey-Nagel) according to the manufacturer’s instructions. We indexed the PCR products for Illumina sequencing by performing a second PCR with nested unique dual index primers using the following program: (1) 95°C for 3 min, (2) 95°C for 30 s, (3) 55°C for 30 s, (4) 72°C for 20 sec, (5) repeat steps 2–4 for 10 cycles, (6) 72°C for 3 min. Afterwards, we assessed the indexed PCR product using gel electrophoresis (1% w/v agarose, 1xTAE buffer), pooled the indexed samples according to band intensity, and subsequently purified the library via AMPure bead cleanup (Beckman Coulter) before proceeding to amplicon sequencing. Samples were sequenced by BMKGENE (Münster, Germany) at 1 Gb output on the Illumina NovaSeq 6000 platform using 150 bp paired-end reads. Subsequently, the reads were demultiplexed and grouped by WISH-tags using mBARq software suite [29]. Misreads or mutations of up to five bases were assigned to the closest correct WISH-tag sequence. The WISH-barcode counts for each mouse in every experiment are available in S1 Table and S2 Table. These counts were used to calculate the relative fitness and Shannon Evenness Index (SEI).

### Calculation of the Shannon Evenness Index

WISH-barcode abundance was determined from amplicon sequencing. For each sample, raw counts were filtered to remove barcodes with fewer than 10 counts to exclude sequencing noise, and the remaining counts were used to calculate the relative abundance (proportion) of each barcode *i* as:


$$\begin{array}{c} p_{i}=\frac{n_{i}}{N} \end{array}$$

where *n*_*i*_ is the read count for barcode *i* and *N* is the total read count across all barcodes detected in that sample.

Shannon diversity (H′) was calculated as:


$$\begin{array}{c} H^{\prime }=-\sum_{i=\text{1}}^{S}p_{i}\text{ln}(p_{i}) \end{array}$$

where *S* is barcode richness, i.e., the number of distinct barcodes detected in the sample (10 WISH-barcodes per inoculation pool).

Shannon evenness was then calculated by normalizing H′ to its theoretical maximum, ln(*S*), which is the value H′ would take if all barcodes were present at equal abundance:


$$\begin{array}{c} J^{\prime }=\frac{H^{\prime }}{H_{max}^{\prime }}=\frac{H^{\prime }}{\text{ln}(S)} \end{array}$$

J′ ranges from 0 to 1, with values approaching 1 indicating that barcodes are represented in near-equal proportions (a polyclonal bottleneck with no dominant lineage), and lower values indicating that a subset of barcodes dominates the population (a narrow bottleneck or strong selective sweep).

### Competitive infection

Streptomycin-pretreated C57BL/6J and 129S6/SvEvTac mice were infected with a 1:1 mixture of *S.* Typhimurium ATCC14028s wild-type and *rsmG* mutant strains, totaling 5 × 10⁷ CFU; both strains carried the P3 plasmid. In a separate set of experiments, mice were infected in the same way with a 1:1 mixture of P3-carrying (Str^+^) and P3-free (Str^-^) ATCC14028s wild type to determine the fitness cost of P3 loss alone. The relative abundance of each strain in the inoculum was enumerated on selective MacConkey agar and used to normalize the bacterial loads recovered on days 1–3 post-infection. The competitive index (CI) was calculated as the normalized ratio of mutant to wild-type CFU. Statistical significance was assessed by comparing the absolute bacterial loads of the two competing strains within each mouse: wild type versus *rsmG*::Kan^R^ mutant, and Str^+^ (P3-carrying) versus Str^-^ (P3-free) wild type.

### Lipocalin-2 analysis of feces samples

Lipocalin-2 levels were measured in fecal samples homogenized in 500 µl PBS using an ELISA assay (DuoSet Lipocalin-2 ELISA kit, DY1857; R&D Systems) according to the manufacturer’s instructions. Fecal pellets were diluted 1:20, 1:400, or left undiluted, and concentrations were determined using Four-Parametric Logistic Regression curve fitting.

### Minimum inhibitory concentration (MIC) determination

MICs were determined by broth microdilution in 96-well microtiter plates. Antibiotics were prepared as sterile stock solutions and serially two-fold diluted in Mueller-Hinton broth 2 to generate the indicated concentration range. Overnight bacterial cultures were diluted into fresh medium and grown to mid-log phase, then adjusted to an inoculum of ~5 × 10^5^ CFU ml^-1^ in the final assay volume. Wells were inoculated with bacteria and included a growth control (no antibiotic) and a sterility control (medium only). Plates were incubated statically at 37 °C for 16–20 h. MIC was defined as the lowest antibiotic concentration that prevented visible growth (no turbidity) compared with the growth control. Where indicated, growth inhibition was additionally quantified by measuring OD_600_ using a plate reader. MIC assays were performed with at least two independent biological replicates, and representative median MICs are reported.

### Single-nucleotide polymorphism (SNP) analysis of ATCC14028s pools

WISH-barcoded ATCC14028s wild-type, Δ*invG*, and Δ*ssaV* strains were grown independently overnight in selective LB medium, followed by genomic DNA isolation using a commercial kit (Qiagen DNeasy Mini Kit) according to the manufacturer’s instructions. Genomic DNA samples were submitted to BMKGENE (Münster, Germany) for whole-genome sequencing. Quality controlled reads were used to assemble genomes with Shovill v1.1.0 (Seemann T (2016) https://github.com/tseemann/shovill) and SKESA v2.5.1 [45] as implemented in Bactopia v3.2.0 [46] using default settings. SNP profiles (and Insertion/Deletions) were generated by mapping quality control reads against a *Salmonella enterica* reference genome. To this end, a reference genome for *Salmonella enterica* subsp. *enterica* serovar Typhimurium str. 14028 (accession CP001363.1 [27]), plasmids P1 (accession HE654724.1), P2 (accession NC_017718.1), and P3 plasmid (accession HE654726.1) was downloaded from NCBI (accessed: 2025.04.15). Snippy v4.6.0 (Seemann T (2015) https://github.com/tseemann/snippy) as implemented in Bactopia was then used to obtain SNP profiles for each strain using default settings against the reference genome and plasmids. Functional annotations of sequences, in which SNPs or insertions/ deletions were detected, were obtained with NCBI Prokaryotic Genome Annotation Pipeline v2025-05–06.build7983 [47]. SNP profiles were aggregated for all strains using Python 3.10.8. Sequencing depths and coverages were calculated with samtools v1.21 [48] and visualized with matplotlib v3.9.1 [49] and seaborn v0.12.2 [50].

### Statistical analysis

No statistical methods were used to predetermine sample sizes. For all mouse experiments, sample sizes of 5 or greater were used, consistent with those reported in previous publications [51]. Data collection and analysis were not performed blind to the conditions of the experiments. Where applicable, the two-tailed Mann-Whitney U test was employed to assess statistical significance between two independent groups (e.g., comparison of the Shannon evenness index between WISH-barcoded wild-type and Δ*invG* pools at day 1 post-infection), as specified in the figure legends. Where paired comparisons within the same animal were performed (e.g., comparing bacterial loads between two strains within the same mouse), the Wilcoxon matched-pairs signed rank test was used instead, as specified in the figure legends. No correction for multiple comparisons was applied; each comparison was treated as an independent hypothesis test, and this is noted as a limitation where relevant. For competitive index data, mice were sampled longitudinally over the 3 days post-infection. Each timepoint was analyzed independently using the two-tailed Mann-Whitney U test to assess between-group differences at that specific timepoint, rather than modeling the trajectory within individual animals; this approach is consistent with prior work from our group [12,14,15,17,37,52] and focuses on cross-sectional differences at biologically relevant timepoints rather than within-animal trends over time. Statistical analyses were performed using GraphPad Prism 10 for Windows. P values were grouped as follows: ns, not significant (P ≥ 0.05); *P < 0.05; **P < 0.01; ***P < 0.001.

## Supporting information

S1 Fig Stable composition of the WISH-barcoded ATCC14028s wild-type, Δ*invG*, and Δ*ssaV* inocula. Relative abundances of individual WISH-barcoded strains within ATCC14028s wild-type (Wt), Δ*invG*, and Δ*ssaV* pools in the inoculum. (**A**) C57BL/6J (SPF, Str) and (**B**) 129S6/SvEvTac (SPF, Str) mice. Each point represents the fraction of an individual barcode relative to total WISH counts per WISH-barcode. The horizontal black line indicates a total fraction of 1. Shannon evenness index (SEI) values and median relative abundances for each WISH-barcoded strain in the respective pool are shown, demonstrating high population evenness and stable pool composition across all three pools.
(TIF)

S2 Fig Whole-genome sequencing depth and coverage of WISH-barcoded ATCC14028s strains. (**A**) Mean sequencing depth per strain across the chromosome (CP001363.1) and plasmids P1, P2, and P3. (**B**) Genome coverage per strain, expressed as the percentage of bases covered by at least one read for the same replicons. Each bar represents an individual WISH-barcoded ATCC14028s strain. While chromosomal and P1 sequencing showed uniformly high depth and near-complete coverage across strains, P2 exhibited low depth and coverage, and P3 showed variable coverage consistent with plasmid loss in a subset of low-abundance strains. P1 and P2 encode complete conjugation machineries and are present at only one to two copies per cell.
(TIF)

S3 Fig Loss of streptomycin resistance confers a competitive fitness cost *in vivo.* (**A, B**) Competitive indices of a Str^-^ strain relative to the Str^+^ wild-type competitor in (**A**) C57BL/6J (n = 9) and (**B**) 129S6/SvEvTac (n = 8) mice at days 1, 2, and 3 post infection. The competitive index (CI) is expressed as the ratio of Str^-^ to Str^+^ colony-forming units recovered from fecal samples. A CI below 1.0 (horizontal line) indicates that the Str^-^ strain is outcompeted by the Str^+^ strain. Each point represents one animal; boxes indicate the median and interquartile range, with whiskers extending to the most extreme values. P values were determined using the Wilcoxon matched-pairs signed rank test (ns, not significant; *P < 0.05; **P < 0.01; ***P < 0.001).
(TIF)

S4 Fig The Δ*invG* and Δ*ssaV* pools exhibit colonization kinetics similar to those of wild-type ATCC14028s. For panels (**A, C, E**) and (**B, D, F**), the respective mouse models are indicated above each row. Panels (**A–D**) represent total *S.* Typhimurium colony-forming units (CFUs) per gram of feces or cecal content (cc), while panels (**E, F**) show Lipocalin-2 levels (ng per gram of feces). Panels (**C, D**) additionally show CFUs recovered from the mesenteric lymph node (mLN), spleen, and liver; LOD denotes the limit of detection. The inflammatory threshold of 500 ng Lipocalin-2 per g feces is indicated by a black line. The sample size (n) for all groups was at least 5, from two independent experiments. Panels (**A-F**) are presented as bar plots, displaying the median and individual data points. P values were determined using the two-tailed Mann–Whitney U test (ns, not significant; *P < 0.05; **P < 0.01; ***P < 0.001).
(TIF)

S1 Table WISH-barcode sequencing read counts in C57BL/6J (SPF, Str) mice. Raw amplicon-sequencing read counts for the ten WISH-barcoded strains of each pool (ATCC14028s wild type, Δ*invG*, Δ*ssaV*) in the inocula (In), in fecal samples collected on days 1–3 post-infection (d1–d3), and in cecal content (cc); each column corresponds to one sample from an individual mouse. Below each block, read counts are converted to relative barcode abundances and used to calculate the Shannon Evenness Index (SEI) as described in Methods. The bottom section summarizes the median relative abundance per barcode in the inocula of all three pools.
(XLSX)

S2 Table WISH-barcode sequencing read counts in 129S6/SvEvTac (SPF, Str) mice. Data are organized as in S1 Table: raw read counts for the ten WISH-barcoded strains of the ATCC14028s wild-type, Δ*invG*, and Δ*ssaV* pools in the inocula (In), fecal samples from days 1–3 post-infection (d1–d3), and cecal content (cc), with the derived relative abundances and Shannon Evenness Index (SEI) below each block.
(XLSX)

S3 Table Whole-genome sequencing SNP analysis of ATCC14028s wild-type WISH-barcoded strains. Single-nucleotide polymorphisms and small insertions/deletions detected in the parental ATCC14028s wild type and the ten WISH-barcoded wild-type strains, identified with Snippy by mapping quality-controlled reads against the *S.* Typhimurium 14028s reference genome (CP001363.1) and plasmids P1–P3. Each row gives the genomic position and reference allele, followed by the variant allele observed in each strain; empty cells indicate identity with the reference. Variant type, predicted effect, locus tag, gene, and product annotation are listed in the right-hand columns.
(XLSX)

S4 Table Whole-genome sequencing SNP analysis of ATCC14028s Δ*invG* WISH-barcoded strains. Variants detected in the Δ*invG* parental strain and the ten WISH-barcoded Δ*invG* strains, called and annotated as described for S3 Table.
(XLSX)

S5 Table Whole-genome sequencing SNP analysis of ATCC14028s Δ*ssaV* WISH-barcoded strains. Variants detected in the Δ*ssaV* parental strain and the ten WISH-barcoded Δ*ssaV* strains, called and annotated as described for S3 Table.
(XLSX)

S6 Table Bacterial strains used in this study. Strain identifier, genotype, method of construction, donor strain where applicable, the name used in the manuscript text, and the source reference. Full citations for the abbreviated references are given on the second sheet.
(XLSX)

S7 Table Oligonucleotide primers used in this study. Primer name, sequence (5′ → 3′), and source. Full citations for the abbreviated references are given on the second sheet.
(XLSX)

S8 Table Plasmids used in this study. Plasmid name, function in this study, and source. Full citations for the abbreviated references are given on the second sheet.
(XLSX)
