# Supplementary material for: WISH-barcoding of Salmonella Typhimurium ATCC14028s strains for population dynamics studies in vivo
Source: PLoS One. 2026 Oct 14;21(10):e0349327. doi: 10.1371/journal.pone.0349327 (PMC13667820; doi:10.1371/journal.pone.0349327)
Supplement: Peer review history

# Peer review: Decision Letter 0

**DOI:** 10.1371/journal.pone.0349327.r001

**Authors:** Mohammad Faezi Ghasemi

27 Jul 2026

Dear Dr. Hardt,

Thank you for submitting your manuscript to PLOS ONE. After careful consideration, we feel that it has merit but does not fully meet PLOS ONE’s publication criteria as it currently stands. Therefore, we invite you to submit a revised version of the manuscript that addresses the points raised during the review process.

Please submit your revised manuscript by Sep 25 2026 11:59PM. If you will need more time than this to complete your revisions, please reply to this message or contact the journal office at plosone@plos.org. When you're ready to submit your revision, log on to https://www.editorialmanager.com/pone/ and select the 'Submissions Needing Revision' folder to locate your manuscript file.

- A letter that responds to each point raised by the academic editor and reviewer(s). You should upload this letter as a separate file labeled 'Response to Reviewers'.
- A marked-up copy of your manuscript that highlights changes made to the original version. You should upload this as a separate file labeled 'Revised Manuscript with Track Changes'.
- An unmarked version of your revised paper without tracked changes. You should upload this as a separate file labeled 'Manuscript'.

If you would like to make changes to your financial disclosure, please include your updated statement in your cover letter. Guidelines for resubmitting your figure files are available below the reviewer comments at the end of this letter.

If applicable, we recommend that you deposit your laboratory protocols in protocols.io to enhance the reproducibility of your results. Protocols.io assigns your protocol its own identifier (DOI) so that it can be cited independently in the future. For instructions see: https://journals.plos.org/plosone/s/submission-guidelines#loc-laboratory-protocols. Additionally, PLOS ONE offers an option for publishing peer-reviewed Lab Protocol articles, which describe protocols hosted on protocols.io. Read more information on sharing protocols at https://plos.org/protocols?utm_medium=editorial-email&utm_source=authorletters&utm_campaign=protocols.

As the corresponding author, your ORCID iD is verified in the submission system and will appear in the published article. PLOS supports the use of ORCID, and we encourage all coauthors to register for an ORCID iD and use it as well. Please encourage your coauthors to verify their ORCID iD within the submission system before final acceptance, as unverified ORCID iDs will not appear in the published article. *Only*  the individual author can complete the verification step; PLOS staff *cannot*  verify ORCID iDs on behalf of authors.

We look forward to receiving your revised manuscript.

Kind regards,

Mohammad Faezi Ghasemi, Ph.D

Academic Editor

PLOS One

Journal Requirements:

When submitting your revision, we need you to address these additional requirements.

1.Please ensure that your manuscript meets PLOS ONE's style requirements, including those for file naming. The PLOS ONE style templates can be found at

https://journals.plos.org/plosone/s/file?id=wjVg/PLOSOne_formatting_sample_main_body.pdf and

https://journals.plos.org/plosone/s/file?id=ba62/PLOSOne_formatting_sample_title_authors_affiliations.pdf

2. To comply with PLOS One submissions requirements, in your Methods section, please provide additional information regarding the experiments involving animals and ensure you have included details on (1) methods of sacrifice, (2) methods of anesthesia and/or analgesia, and (3) efforts to alleviate suffering.

3. Please note that funding information should not appear in any section or other areas of your manuscript. We will only publish funding information present in the Funding Statement section of the online submission form. Please remove any funding-related text from the manuscript.

4. We note that the grant information you provided in the ‘Funding Information’ and ‘Financial Disclosure’ sections do not match.

When you resubmit, please ensure that you provide the correct grant numbers for the awards you received for your study in the ‘Funding Information’ section.

5. Thank you for stating the following financial disclosure:

“This work has been funded by grants from the Swiss National Science Foundation (310030_192567, 10.001.588 and NCCR Microbiomes grant 51NF40_180575) to W.-D.H.”

Please state what role the funders took in the study.  If the funders had no role, please state: "The funders had no role in study design, data collection and analysis, decision to publish, or preparation of the manuscript."

If this statement is not correct you must amend it as needed.

Please include this amended Role of Funder statement in your cover letter; we will change the online submission form on your behalf.

6. Please expand the acronym “NCCR” (as indicated in your financial disclosure) so that it states the name of your funders in full.

This information should be included in your cover letter; we will change the online submission form on your behalf.

7. Please note that your Data Availability Statement is currently missing the direct link to access each database. If your manuscript is accepted for publication, you will be asked to provide these details on a very short timeline. We therefore suggest that you provide this information now, though we will not hold up the peer review process if you are unable.

8. Please include captions for your Supporting Information files at the end of your manuscript, and update any in-text citations to match accordingly. Please see our Supporting Information guidelines for more information: http://journals.plos.org/plosone/s/supporting-information.

9. If the reviewer comments include a recommendation to cite specific previously published works, please review and evaluate these publications to determine whether they are relevant and should be cited. There is no requirement to cite these works unless the editor has indicated otherwise.

Additional Editor Comments:

Major Concerns

Clarity of the ΔinvG and ΔssaV Backgrounds

The manuscript does not adequately explain *why*  the ΔinvG and ΔssaV mutants were selected as comparator strains. While the rationale is partially explained (invG is required for invasion, ssaV for systemic survival), the justification for using these specific mutants to demonstrate the *methodological*  issue is not fully developed.

Strengthen the introduction to explicitly state that these mutants were chosen because they represent well-characterized genetic backgrounds with distinct virulence phenotypes that would be expected to affect different stages of infection. This helps contextualize why their stable colonization patterns are informative.

Incomplete Discussion of the Mechanism Underlying Background-Specific Effects

The authors note that "this selective pressure appeared to be specific to the wild-type background, though the underlying reasons for this remain unclear" (lines 210-211). While acknowledging uncertainty is appropriate, the discussion could be strengthened by exploring potential hypotheses more thoroughly.

Expand the discussion to include plausible explanations for why ΔinvG and ΔssaV backgrounds did not acquire rsmG mutations or lose P3. Consider:

Potential fitness costs of losing P3 being more severe in mutant backgrounds

Differences in growth rates affecting mutation accumulation

Possibility of compensatory mutations in mutant backgrounds that were not examined

Statistical Analysis Details

The statistical methods section is somewhat brief and lacks some details about multiple comparison corrections.

Specify whether multiple comparison corrections (e.g., Bonferroni, Holm-Sidak) were applied for the SEI comparisons shown in Figure 2

Clarify which specific comparisons were made using the Mann-Whitney U test

Consider whether repeated measures analysis would be appropriate for the longitudinal data

Limited Discussion of the Transposon Sequencing Context

The introduction mentions RB-TnSeq but does not fully contextualize how WISH-barcoding complements or differs from this approach in terms of experimental design and interpretation.

Expand the introduction to briefly discuss how the population stability issues identified here might affect interpretation of both WISH-barcoding and RB-TnSeq experiments, noting that the findings likely have broader implications beyond just WISH-barcoding.

Data Accessibility

The authors have made sequencing data available under accession number PRJEB85516 and code available on GitHub. The manuscript states "all samples, except for organs, were enriched in selective LB" (lines 81-82). It would be helpful to explicitly state why organs were excluded from enrichment.

Minor Issues

Abstract

Consider adding a brief statement about the broader implications for the field.

Introduction

The introduction effectively establishes the rationale for the study.

Lines 46-49: The explanation of RB-TnSeq could be slightly more precise about the randomness of transposon insertion being a drawback for hypothesis-driven studies.

Results

Figure 1: Well-designed and clearly illustrates the experimental workflow.

The separation of strains into high and low fitness groups is compelling.

Consider adding a panel showing the inoculum SEI values to emphasize that the population instability arose during strain construction, not during the experiment itself.

The fact that the same pre-mixed cryostocks were used for both mouse models (lines 119-121) is an important control—this point could be emphasized more prominently.

Lines 173-174: "all strains exceeded the established inflammatory threshold of 500 ng/g feces." This threshold was established in a previous study—please confirm that this reference is appropriate and that the same ELISA methodology was used.

Line 271: "Figure legends" section appears truncated—consider reformatting to ensure all legend details are complete.

Supplementary Data: The supplementary data are extensive and appropriate. Consider adding a brief description of the supplementary data structure in the main text to guide readers.

Lines 229-231: The reference to "inflammation-associated bottlenecks" in SL1344 is valuable context. Expand slightly to explain how these bottlenecks differ mechanistically from the stability issues observed here (stochastic loss vs. mutation-driven stratification).

With regards,

[Note: HTML markup is below. Please do not edit.]

Reviewers' comments:

Reviewer's Responses to Questions

**Comments to the Author**

1. Is the manuscript technically sound, and do the data support the conclusions?

Reviewer #1: Partly

Reviewer #2: Yes

**********

2. Has the statistical analysis been performed appropriately and rigorously?

Reviewer #1: No

Reviewer #2: Yes

**********

3. Have the authors made all data underlying the findings in their manuscript fully available?

The PLOS Data policy

Reviewer #1: Yes

Reviewer #2: Yes

**********

4. Is the manuscript presented in an intelligible fashion and written in standard English?

Reviewer #1: Yes

Reviewer #2: Yes

**********

Reviewer #1: Manuscript Number: PONE-D-26-20998 Title: WISH-barcoding of Salmonella Typhimurium dynamics studies in vivo

Summary of the Research

The manuscript by Schubert et al. evaluates the implementation of WISH-barcoded Salmonella Typhimurium ATCC14028s pools to investigate bacterial population dynamics during infection. Utilizing a Streptomycin-pretreated mouse model, the authors demonstrate that wild-type barcoded pools exhibit uneven population structures that are characterized by stochastic strain loss and unequal subpopulation expansion. Through whole-genome sequencing, the study identifies that these low-fitness strains frequently harbor mutations in the 16S rRNA methyltransferase gene rsmG or have lost the P3 plasmid. The authors conclude that rsmG mutations are inadvertently enriched during in vitro construction under streptomycin selection, and they establish that these mutations, along with P3 plasmid loss, significantly attenuate gut luminal colonization.

The study provides a highly relevant cautionary framework for molecular microbiologists, highlighting the intense selective pressures introduced during library preparation and genome editing. However, several methodological and analytical aspects require further clarification before the manuscript can be considered suitable for publication.

Major Comments

While the manuscript successfully identifies critical bottlenecks in utilizing the P3 plasmid for in vitro pool construction, the following scientific issues should be addressed to ensure the rigor expected by PLOS ONE:

Mechanism of rsmG Selection: The authors note that rsmG mutations are enriched during strain construction involving streptomycin selection, following the loss of the P3 plasmid. The manuscript should expand on the regulatory consequences of this mutation, specifically whether the absence of this 16S rRNA methyltransferase strictly confers a survival advantage during molecular cloning, or if it triggers broader transcriptomic shifts that inherently limit colonization fitness independent of the barcoding context.

Plasmid P3 Stability Baseline: The loss of the P3 plasmid is cited as a primary driver of in vivo attenuation. The authors should quantify the baseline stability and spontaneous curing rate of this plasmid in the ATCC14028s strain under standard laboratory conditions without antibiotic pressure to better contextualize the in vitro construction risks.

In Vivo Model Nuances: The experimental design utilizes two distinct mouse lineages: C57BL/6J and 129S6/SvEvTac. Differences in the host genetic background (and potentially baseline microbiota) should be explicitly discussed in relation to the Shannon Evenness Index data presented in Figure 2, as bottleneck severity can vary between these models.

Virulence Factor Controls: The inclusion of ΔinvG and ΔssaV mutant pools serves as a robust control for SPI-1 and SPI-2 dependencies during infection. The manuscript must clarify if the same rate of rsmG mutation enrichment and P3 loss was observed in these specific virulence-deficient backgrounds compared to the wild-type pools during their respective in vitro construction phases.

Minor Comments

Data Availability Transparency: The manuscript states that whole-genome sequencing data will be made available at the European Nucleotide Archive. The authors must ensure that the specific accession number provided (PRJEB81516) is fully accessible without restriction prior to final publication, per journal policy.

Statistical Clarity in Figures: In Figure 3, the competitive index (rmsG::Kan/wild type) data plots display various significance markers across different days post-infection. The specific statistical tests applied to account for these unevenly distributed populations must be transparently detailed in the statistical analysis methods section and figure legends.

Figure Formatting: The minimum inhibitory concentration (MIC) heatmap in Figure 3b should clearly define the biological replicates utilized to calculate the displayed values. Additionally, ensure the specific optical density thresholds used for the OD600nm scale are explicitly stated in the legend.

Typographical and Formatting Errors: There are minor spacing issues and fragmented text in the abstract and reference sections that must be corrected to ensure strict adherence to typesetting guidelines.

Recommendation: Major Revision

Reviewer #2: I read this manuscript with interesting that presents a valuable contribution to the field of microbiology and pathogen dynamics. It addresses an important area of research by adapting WISH-barcoding for Salmonella ATCC14028s. This method could significantly enhance our understanding of pathogen population dynamics during infections, particularly in the context of streptomycin-pretreated mouse models.

I have a minor comment that could be avoided in the later study.

In fig. 2, the authors declaimed that WISH-barcodes with a high relative abundance in the C57BL/6J model also exhibited a high relative abundance in the 129S6/SvEvTac model.

Is there a possibility that this strain exhibits an unknown mutation, resulting in enhanced host gut adaptability (i.e., W115?)? Alternatively, does this barcode exhibit high efficiency during the two rounds of PCR?

The authors also declaimed that We reasoned that this was likely attributable to mutations acquired during the construction of the WISH-barcoded ATCC14028s wild-type strains. It that, we would removed these unexpected barcode-WT to establish a stable pre-inoculation pools.

The authors did not reconstruct a wild-type strain pool validated, i.e., retaining P3 and maintaining intact rsmG, nor did they demonstrate that its SEI and barcode abundance remained stable in mice.

Moreover, please ensure that all figures are clear enough.

**********

what does this mean?). If published, this will include your full peer review and any attached files.

If you choose “no”, your identity will remain anonymous but your review may still be made public.

**Do you want your identity to be public for this peer review?**  For information about this choice, including consent withdrawal, please see our Privacy Policy

Reviewer #1: No

Reviewer #2: No

**********

[NOTE: If reviewer comments were submitted as an attachment file, they will be attached to this email and accessible via the submission site. Please log into your account, locate the manuscript record, and check for the action link "View Attachments". If this link does not appear, there are no attachment files.]

To ensure your figures meet our technical requirements, please review our figure guidelines: https://journals.plos.org/plosone/s/figures

You may also use PLOS’s free figure tool, NAAS, to help you prepare publication quality figures: https://journals.plos.org/plosone/s/figures#loc-tools-for-figure-preparation.

NAAS will assess whether your figures meet our technical requirements by comparing each figure against our figure specifications.

# Author comment: Author response to Decision Letter 1

**DOI:** 10.1371/journal.pone.0349327.r002

19 Aug 2026

Point-by-point responses to the reviewers’ comments

Editor comments:

Major Concerns

1. Clarity of the ΔinvG and ΔssaV Backgrounds

The manuscript does not adequately explain why the ΔinvG and ΔssaV mutants were selected as comparator strains. While the rationale is partially explained (invG is required for invasion, ssaV for systemic survival), the justification for using these specific mutants to demonstrate the methodological issue is not fully developed.

Strengthen the introduction to explicitly state that these mutants were chosen because they represent well-characterized genetic backgrounds with distinct virulence phenotypes that would be expected to affect different stages of infection. This helps contextualize why their stable colonization patterns are informative.

Author response: Thank you for pointing out this problem. To provide a clearer statement, we have extended the Introduction to include our reasoning for including invG and ssaV.

(L76-L77)

In addition to the wild-type strain, we analyzed invG- and ssaV-deficient ATCC14028s WISH-barcoded pools. InvG is a structural component of the Type III Secretion System 1 (T3SS-1) and is essential for host cell invasion [1, 2], whereas SsaV is a core component of T3SS-2, which is required for intracellular survival and systemic dissemination [3, 4]. Traversing the tissue barrier constitutes a population bottleneck, the effects of which can extend to the gut-luminal population during reseeding by tissue-lodged Salmonella. By comparing ΔinvG and ΔssaV mutants with the wild-type pool, we assessed whether defects in epithelial invasion or systemic survival alter S. Typhimurium population stability in the streptomycin-pretreated mouse model during gut-luminal colonization.

(bold text was added)

2. Incomplete Discussion of the Mechanism Underlying Background-Specific Effects

The authors note that "this selective pressure appeared to be specific to the wild-type background, though the underlying reasons for this remain unclear" (lines 210-211). While acknowledging uncertainty is appropriate, the discussion could be strengthened by exploring potential hypotheses more thoroughly.

Expand the discussion to include plausible explanations for why ΔinvG and ΔssaV backgrounds did not acquire rsmG mutations or lose P3. Consider:

Potential fitness costs of losing P3 being more severe in mutant backgrounds

Differences in growth rates affecting mutation accumulation

Possibility of compensatory mutations in mutant backgrounds that were not examined

Author response: Thank you for these interesting points to consider. Since we normalize the inocula to OD₆₀₀ of 2, we also measured the density after the 4 h subculture. This provides us with an indicator for differences in growth rates of the different strains. We never observed any severe growth defect in the ΔinvG or ΔssaV mutants; for this reason, we do not believe that growth-rate-dependent differences in mutation accumulation account for this strain-specific selective pressure. Furthermore, whole-genome sequencing confirmed that the ΔinvG and ΔssaV pools still contain the P3 plasmid, and we did not observe any compensatory mutations in these backgrounds, consistent with the absence of rsmG-related SNPs in the first place.

To address the argument about growth rate, we have included the following sentence in the Discussion:

(L214-L220)

This selective pressure appeared to be specific to the wild-type background, though the underlying reasons for this remain unclear. Notably, wild-type, ΔinvG, and ΔssaV strains grow with comparable rates in LB during routine inoculum preparation, making growth-rate-dependent differences in mutation accumulation an unlikely explanation for this strain-specific selective pressure. Furthermore, all WISH barcodes used in this study were previously employed in our studies with S. Typhimurium SL1344 [5-10], in which no fitness effects attributable to specific barcodes were observed. We therefore consider it unlikely that WISH barcode identity itself influences Salmonella strain fitness in our experimental system.

(bold text was added)

3. Statistical Analysis Details

The statistical methods section is somewhat brief and lacks some details about multiple comparison corrections.

Specify whether multiple comparison corrections (e.g., Bonferroni, Holm-Sidak) were applied for the SEI comparisons shown in Figure 2

Clarify which specific comparisons were made using the Mann-Whitney U test

Consider whether repeated measures analysis would be appropriate for the longitudinal data

Author response: We thank the reviewer for these constructive suggestions and have expanded the Statistical Analysis section accordingly. We would like to clarify that no correction for multiple comparisons (e.g., Bonferroni, Holm-Šídák) was applied in this study; each pairwise comparison shown in Figure 2 and elsewhere was treated as an independent test using the two-tailed Mann-Whitney U test, and we have now stated this explicitly rather than leaving it implicit.

We amended the Statistical Analysis section accordingly:

(L506-L520)

No statistical methods were used to predetermine sample sizes. For all mouse experiments, sample sizes of 5 or greater were used, consistent with those reported in previous publications [11]. Data collection and analysis were not performed blind to the conditions of the experiments. Where applicable, the two-tailed Mann-Whitney U test was employed to assess statistical significance between two independent groups (e.g., comparison of the Shannon evenness index between WISH-barcoded wild-type and ΔinvG pools at day 1 post-infection), as specified in the figure legends. Where paired comparisons within the same animal were performed (e.g., comparing bacterial loads between two strains within the same mouse), the two-tailed Wilcoxon signed-rank test was used instead, as specified in the figure legends. No correction for multiple comparisons was applied; each comparison was treated as an independent hypothesis test, and this is noted as a limitation where relevant. For competitive index data, mice were sampled longitudinally over the 3 days post-infection. Each timepoint was analyzed independently using the two-tailed Mann-Whitney U test to assess between-group differences at that specific timepoint, rather than modeling the trajectory within individual animals; this approach is consistent with prior work from our group [5-9, 12] and focuses on cross-sectional differences at biologically relevant timepoints rather than within-animal trends over time. Statistical analyses were performed using GraphPad Prism 10 for Windows. P values were grouped as follows: ** ≙ P < 0.005; * ≙ P < 0.05; ns ≙ P > 0.05.

(bold text was added)

4. Limited Discussion of the Transposon Sequencing Context

The introduction mentions RB-TnSeq but does not fully contextualize how WISH-barcoding complements or differs from this approach in terms of experimental design and interpretation.

Expand the introduction to briefly discuss how the population stability issues identified here might affect interpretation of both WISH-barcoding and RB-TnSeq experiments, noting that the findings likely have broader implications beyond just WISH-barcoding.

Author response: Thank you for pointing this out. We have included the following statement to elaborate on the advantage of WISH-barcoding — namely, that it relies on a smaller number of strains. Thereby, the population structures are typically less affected by population bottlenecks compared to BR-TnSeq experiments, where we use up to 2000 strains per pool:

(L47-L48)

Transposon mutagenesis works by randomly inserting a transposon into the genome to disrupt genes, allowing identification of genes required for fitness or survival by tracking which insertions are lost under selective conditions [13]. This randomness can be a drawback when focusing on a specific physiological process, such as nutrient utilization, particularly when immune responses impose pronounced bottlenecks on the pathogen population — a limitation for approaches relying on large numbers of mutant strains, such as RB-TnSeq [5, 14].

(bold text was added)

5. Data Accessibility

The authors have made sequencing data available under accession number PRJEB85516 and code available on GitHub. The manuscript states "all samples, except for organs, were enriched in selective LB" (lines 81-82). It would be helpful to explicitly state why organs were excluded from enrichment.

Author response: Thank you for this reminder. All of the sequencing data will be made available prior to publication. Regarding organ sampling, previous barcoding experiments have shown that traversal from the gut lumen to systemic sites such as the spleen imposes a population bottleneck, resulting in a loss of WISH barcode diversity. For this reason, we opted not to assess WISH barcodes in organ samples.

Minor Issues

6. Abstract

Consider adding a brief statement about the broader implications for the field.

Author response: Thank you for this comment. We have added a sentence on the broader implications for the field:

(L27-L29)

While population dynamics experiments with ATCC 14028s are feasible in principle, wild-type strains are prone to acquiring fitness-altering mutations during in vitro construction when using the P3 plasmid and streptomycin, highlighting the need for careful pool validation prior to use and for minimizing antibiotic exposure during strain construction when the strain of interest has no natural resistance, to reduce the risk of selecting mutations.

(bold text was added)

7. Introduction

The introduction effectively establishes the rationale for the study.

Lines 46-49: The explanation of RB-TnSeq could be slightly more precise about the randomness of transposon insertion being a drawback for hypothesis-driven studies.

Author response: Thank you, we adapted the sentence and included “for hypothesis-driven studies”

8. Results

Figure 1: Well-designed and clearly illustrates the experimental workflow.

The separation of strains into high and low fitness groups is compelling.

Consider adding a panel showing the inoculum SEI values to emphasize that the population instability arose during strain construction, not during the experiment itself.

Author response: Thank you for this point. We have included a figure showing strain abundance and the corresponding Shannon evenness index (SEI) in the inoculum in Supplementary Figure 1. We also included a section in the material and methods section to explicitly specify how me calculated the SEI:

(L446-L461)

Calculation of the Shannon Evenness Index

WISH-barcode abundance was determined from amplicon sequencing. For each sample, raw counts were filtered to remove barcodes with fewer than 10 counts to exclude sequencing noise, and the remaining counts were used to calculate the relative abundance (proportion) of each barcode i as:

p_i=n_i/N

where ni is the read count for barcode i and N is the total read count across all barcodes detected in that sample.

Shannon diversity (H′) was calculated as:

H^'=-∑_(i=1)^S▒p_i ln⁡(p_i)

where S is barcode richness, i.e. the number of distinct barcodes detected in the sample (10 WISH barcodes per inoculation pool).

Shannon evenness was then calculated by normalising H′ to its theoretical maximum, ln(S), which is the value H′ would take if all barcodes were present at equal abundance:

J^'=H^'/(H_max^' )=H^'/(ln⁡(S))

J′ ranges from 0 to 1, with values approaching 1 indicating that barcodes are represented in near-equal proportions (a polyclonal bottleneck with no dominant lineage), and lower values indicating that a subset of barcodes dominates the population (a narrow bottleneck or strong selective sweep).

9. The fact that the same pre-mixed cryostocks were used for both mouse models (lines 119-121) is an important control—this point could be emphasized more prominently.

Author response: Thank you for pointing this important aspect out. For this reason we added the following sentence:

(L125-L126)

Interestingly, WISH-barcodes with a high relative abundance in the C57BL/6J model also exhibited a high relative abundance in the 129S6/SvEvTac model, whereas low-abundance WISH-barcodes showed noticeably more pronounced mouse-to-mouse variability (Fig 2D). Importantly, all inocula exhibited SEI values >0.95, with each WISH-barcoded strain contributing approximately 9.5% of the total population in both mouse models (S1 Fig A, B). Both experiments were performed independently using the same pre-mixed WISH-barcoded ATCC14028s cryostocks (see Methods).

(bold text was added)

10. Lines 173-174: "all strains exceeded the established inflammatory threshold of 500 ng/g feces." This threshold was established in a previous study—please confirm that this reference is appropriate and that the same ELISA methodology was used.

Author response: Indeed, in this case, an ELISA-based methodology was also used.

11. Line 271: "Figure legends" section appears truncated—consider reformatting to ensure all legend details are complete.

Author response: Thank you for pointing this out. We have double-checked all figure legends to ensure they are complete.

12. Supplementary Data: The supplementary data are extensive and appropriate. Consider adding a brief description of the supplementary data structure in the main text to guide readers.

Author response: We have added a Contents sheet as the first sheet of the Excel spreadsheet and also included a separate section in the main text:

Supplementary Data

S1 Table. WISH-barcode sequencing read counts in C57BL/6J (SPF, Str) mice.

S2 Table. WISH-barcode sequencing read counts in 129S6/SvEvTac (SPF, Str) mice.

S3 Table. Whole-genome sequencing SNP analysis of ATCC14028s wild-type WISH-barcoded strains.

S4 Table. Whole-genome sequencing SNP analysis of ATCC14028s ΔinvG WISH-barcoded strains.

S5 Table. Whole-genome sequencing SNP analysis of ATCC14028s ΔssaV WISH-barcoded strains.

S6 Table. Bacterial strains used in this study.

S7 Table. Oligonucleotide primers used in this study.

S8 Table. Plasmids used in this study.

S1 Fig. Stable composition of the WISH-barcoded ATCC14028s wild-type, ΔinvG, and ΔssaV inocula.

S2 Fig. Whole-genome sequencing depth and coverage of WISH-barcoded ATCC14028s strains.

S3 Fig. Loss of streptomycin resistance confers a competitive fitness cost in vivo.

S4 Fig. The ΔinvG and ΔssaV pools exhibit colonization kinetics similar to those of wild-type ATCC14028s

13. Lines 229-231: The reference to "inflammation-associated bottlenecks" in SL1344 is valuable context. Expand slightly to explain how these bottlenecks differ mechanistically from the stability issues observed here (stochastic loss vs. mutation-driven stratification).

Author response: Thank you for providing this excellent wording. We have incorporated it into the following section:

(L249)

The mutation-driven stratification of the wild-type ATCC14028s pool into low- and high-fitness subpopulations is attributable to the inadvertent loss of P3 plasmid and acquisition of rsmG mutations during strain construction, both of which imposed a fitness cost during in vivo colonization.

(bold text was added)

With regards,

Mohammad Faezi Ghasemi, Ph.D

Academic Editor

PLOS One

Reviewers' comments:

Reviewer's Responses to Questions

Reviewer #1: Manuscript Number: PONE-D-26-20998 Title: WISH-barcoding of Salmonella Typhimurium dynamics studies in vivo

Summary of the Research

The manuscript by Schubert et al. evaluates the implementation of WISH-barcoded Salmonella Typhimurium ATCC14028s pools to investigate bacterial population dynamics during infection. Utilizing a Streptomycin-pretreated mouse model, the authors demonstrate that wild-type barcoded pools exhibit uneven population structures that are characterized by stochastic strain loss and unequal subpopulation expansion. Through whole-genome sequencing, the study identifies that these low-fitness strains frequently harbor mutations in the 16S rRNA methyltransferase gene rsmG or have lost the P3 plasmid. The authors conclude that rsmG mutations are inadvertently enriched during in vitro construction under streptomycin selection, and they establish that these mutations, along with P3 plasmid loss, significantly attenuate gut luminal colonization.

The study provides a highly relevant cautionary framework for molecular microbiologists, highlighting the intense selective pressures introduced during library preparation and genome editing. However, several methodological and analytical aspects require further clarification before the manuscript can be considered suitable for publication.

Author response: Thank you for this positive assessment of our paper and for the time you took to provide comments.

Major Comments

1. While the manuscript successfully identifies critical bottlenecks in utilizing the P3 plasmid for in vitro pool construction, the following scientific issues should be addressed to ensure the rigor expected by PLOS ONE:

a) Mechanism of rsmG Selection: The authors note that rsmG mutations are enriched during strain construction involving streptomycin selection, following the loss of the P3 plasmid. The manuscript should expand on the regulatory consequences of this mutation, specifically whether the absence of this 16S rRNA methyltransferase strictly confers a survival advantage during molecular cloning, or if it triggers broader transcriptomic shifts that inherently limit colonization fitness independent of the barcoding context.

Author response: We thank the reviewer for raising this important mechanistic point. We agree that distinguishing between a direct selective advantage during cloning and a broader, colonization-relevant regulatory consequence is important, and we have expanded on this in the revised manuscript.

Mechanistically, RsmG is a 16S rRNA methyltransferase that modifies G527 in helix 18 of the ribosomal decoding site. Loss of this methylation reduces streptomycin binding affinity at the decoding site, conferring low-level streptomycin resistance. Under the streptomycin selection pressure applied during strain construction, this creates a direct and strong survival advantage for spontaneous rsmG mutants, independent of any effect on general fitness i.e., rsmG loss is selected for because it directly antagonizes the selective agent, not because it improves fitness more broadly.

Since RsmG is a methyltransferase rather than a direct virulence or colonization factor, any effect of its loss on fitness would be indirect, mediated through alterations to the gene expression pattern of Salmonella rather than a direct effect on colonization. However, deciphering the precise mechanism by which RsmG-mediated rRNA methylation shapes the transcriptome or proteome is beyond the scope of this study.

(L251-L254)

We added the following sentence to the discussion:

Given its role as a methyltransferase rather than a direct virulence or colonization factor, RsmG most likely affects fitness indirectly, through altered ribosome function and translational fidelity. Deciphering the precise molecular basis of this fitness cost, however, is beyond the scope of this study.

(bold text was added)

b) Plasmid P3 Stability Baseline: The loss of the P3 plasmid is cited as a primary driver of in vivo attenuation. The authors should quantify the baseline stability and spontaneous curing rate of this plasmid in the ATCC14028s strain under standard laboratory conditions without antibiotic pressure to better contextualize the in vitro construction risks.

Author response: We thank the reviewer for raising this point. While we agree that a systematic quantification of baseline P3 stability and spontaneous curing rate in ATCC14028s under antibiotic-free conditions would be an interesting addition, we consider this analysis to be beyond the scope of the present study. Our rationale for transforming ATCC14028s with P3 was to render it streptomycin-resistant, thereby reducing its susceptibility to antibiotic-mediated killing in the streptomycin-pretreated mouse model. However, since streptomycin exposure can lead to rsmG mutations not only in ATCC 14028s but also in other species [15, 16], we would refrain from using streptomycin-dependent models for ATCC 14028s in the future.

2. In Vivo Model Nuances: The experimental design utilizes two distinct mouse lineages: C57BL/6J and 129S6/SvEvTac. Differences in the host genetic background (and potentially baseline microbiota) should be explicitly discussed in relation to the Shannon Evenness Index data presented in Figure 2, as bottleneck severity can vary between these models.

Author response: Thank you for pointing this out. Regarding microbiota differences: these are negligible, as high-dose streptomycin pretreatment depletes the microbiota and renders both mouse lines susceptible to Salmonella infection. Regarding host genetic background: the most consequential difference for Salmonella infection is Nramp1 (Slc11a1), which is functional in 129S6/SvEvTac but carries a loss-of-function mutation in C57BL/6J. NRAMP1 is a phagosomal-membrane transporter that effluxes Fe²⁺ and Mn²⁺, restricting the availability of these divalent metals to intracellular Salmonella; its functional presence in 129S6/SvEvTac confers substantially better control of systemic infection compared with C57BL/6J. This difference is highlighted in the Introduction.

(L69-L72)

We added the following sentence in the introduction:

NRAMP1 is a phagosomal-membrane transporter that effluxes Fe²⁺ and Mn²⁺, restricting the availability of these divalent metals to intracellular Salmonella; its functional presence in 129S6/SvEvTac confers substantially better control of systemic infection compared with C57BL/6J [17].

(bold text was added)

3. Virulence Factor Controls: The inclusion of ΔinvG and ΔssaV mutant pools serves as a robust control for SPI-1 and SPI-2 dependencies during infection. The manuscript must clarify if the same rate of rsmG mutation enrichment and P3 loss was observed in these specific virulence-deficient backgrounds compared to the wild-type pools during their respective in vitro construction phases.

Author response: Thank you for this suggestion. Indeed, assessing rsmG mutant enrichment and P3 loss in the invG and ssaV backgrounds would be an important piece of data. We have therefore addressed this point in Supplementary Figure 2, showing that all strains in the invG and ssaV pools carry P3, while none show any SNPs in rsmG (Supplementary Data 4 and 5).

Minor Comments

4. Data Availability Transparency: The manuscript states that whole-genome sequencing data will be made available at the European Nucleotide Archive. The authors must ensure that the specific accession number provided (PRJEB81516) is fully accessible without restriction prior to final publication, per journal policy.

Author response: Thank you for this reminder. All of the sequencing data will be made available prior to publication.

5. Statistical Clarity in Figures: In Figure 3, the competitive index (rmsG::Kan/wild type) data plots display various significance markers across different days post-infection. The specific statistical tests applied to account for these unevenly distributed populations must be transparently detailed in the statistical analysis methods section and figure legends.

Author response: Thank you for pointing out this inaccuracy. For a more precise description of the statistical analysis, we adapted the following sentence:

(L334-L336)

Statistical significance was assessed using the Wilcoxon matched-pairs signed rank test between wild-type and rsmG::KanR mutant bacterial loads in the respective mouse model (ns, not significant; *P < 0.05; **P < 0.01; ***P < 0.001).

(bold was added)

6. Figure Formatting: The minimum inhibitory concentration (MIC) heatmap in Figure 3b should clearly define the biological replicates utilized to calculate the displayed values. Additionally, ensure the specific optical density thresholds used for the OD600nm scale are explicitly stated in the legend.

Author response: Thank you for this comment. To increase transparency, we have included a sentence specifying the number of biological replicates:

(L328-L331)

Minimal inhibitory concentration (MIC) assay for streptomycin showing growth (OD₆₀₀) of wild type, wild type rendered streptomycin resistant with P3 plasmid (Wt Strᴿ), rsmG mutant, and rsmG mutant with P3 plasmid across increasing streptomycin concentrations. MIC was defined as the lowest concentration at which OD₆₀₀ remained within 0.2 of the no-antibiotic growth-inhibited baseline (i.e., medium-only level), clearly separated from the OD₆₀₀ ≥ ~0.8 seen in wells with visible growth. MIC assays were performed with at least two independent biological replicates, and representative median MICs are reported.

(bold was added)

7. Typographical and Formatting Errors: There are minor spacing issues and fragmented text in the abstract and reference sections that must be corrected to ensure strict adherence to typesetting guidelines.

Author response: Thank you for reminding us. To reduce typographical and formatting errors, we have thoroughly proofread and corrected the manuscript prior to resubmission.

Recommendation: Major Revision

Reviewer #2: I read this manuscript with interesting that presents a valuable contribution to the field of microbiology and pathogen dynamics. It addresses an important area of research by adapting WISH-barcoding for Salmonella ATCC14028s. This method could significantly enhance our understanding of pathogen population dynamics during infections, particularly in the context of streptomycin-pretreated mouse models.

1. I have a minor comment that could be avoided in the later study.

In fig. 2, the authors declaimed that WISH-barcodes with a high relative abundance in the C57BL/6J model also exhibited a high relative abundance in the 129S6/SvEvTac model.

Is there a possibility that this strain exhibits an unknown mutation, resulting in enhanced host gut adaptability (i.e., W115?)? Alternatively, does this barcode exhibit high efficiency during the two rounds of PCR?

Author response: Thank you for this interesting question. All of the WISH barcodes used in this study were also used in our previous studies on SL1344 [5-8]. In none of these studies did we observe a fitness effect of specific WISH barcodes, such as W115. For this reason, we would exclude an effect of WISH barcodes on Salmonella strain fitness.

We added the following section to the discussion:

(L216-L220)

Furthermore, all WISH barcodes used in this study were previously employed in our studies with S. Typhimurium SL1344 [5-10], in which no fitness effects attributable to specific barcodes were observed. We therefore consider it unlikely that WISH barcode identity itself influences Salmonella strain fitness in our experimental system.

(bold text was added)

2. The authors also declaimed that We reasoned that this was likely attributable to mutations acquired during the construction of the WISH-barcoded ATCC14028s wild-type strains. It that, we would removed these unexpected barcode-WT to establish a stable pre-inoculation pools.

The authors did not reconstruct a wild-type strain pool validated, i.e., retaining P3 and maintaining intact rsmG, nor did they demonstrate that its SEI and barcode abundance remained stable in mice.

Author response: Thank you for pointing this out. We have used WISH-barcoding in a wide range of species [10], including extensively in the S. Typhimurium strains SL1344 and ATCC 14028s [5-8]. We never observed this behaviour in any of the aforementioned examples except ATCC14028s. We can therefore be fairly confident that this is ATCC14028s-specific, rather than a problem with our WISH barcodes. Indeed, we already have experimental data showing 5 stable WISH-barcoded ATCC14028s wild-type strains that retain P3 and do not acquire a mutation in rsmG (Fig 2C, D).

We have added a sentence to the discussion section, as outlined in our response to comment 2 above.

References

1. Barthel M, Hapfelmeier S, Quintanilla-MartíNez L, Kremer M, Rohde M, Hogardt M, et al. Pretreatment of Mice with Streptomycin Provides a<i>Salmonella enterica Serovar Typhimurium Colitis Model That Allows Analysis of Both Pathogen and Host. Infection and Immunity. 2003;71(5):2839-58. doi: 10.1128/iai.71.5.2839-2858.2003.

2. Watson PR, Galyov EE, Paulin SM, Jones PW, Wallis TS. Mutation of invH, but not stn, reduces Salmonella-induced enteritis in cattle. Infection and immunity. 1998;66(4):1432-8.

3. Hensel M. Salmonella pathogenicity island 2. Molecular microbiology. 2000;36(5):1015-23.

4. Waterman SR, Holden DW. Functions and effectors of the Salmonella pathogenicity island 2 type III secretion system. Cellular microbiology. 2003;5(8):501-11.

5. Schubert C, Nguyen BD, Sichert A, Napflin N, Sintsova A, Feer L, et al. Monosaccharides drive Salmonella gut colonization in a context-dependent or -independent manner. Nat Commun. 2025;16(1):1735. Epub 2025/02/19. doi: 10.1038/s41467-025-56890-y. PubMed PMID: 39966379.

6. Laganenka L, Schubert C, Sichert A, Kalita I, Barthel M, Nguyen BD, et al. Interplay between chemotaxis, quorum sensing, and metabolism regulates Escherichia coli-Salmonella Typhimurium interactions in vivo. PLoS Pathog. 2025;21(5):e1013156. Epub 2025/05/02. doi: 10.1371/journal.ppat.1013156. PubMed PMID: 40315408; PubMed Central PMCID: PMCPMC12074654.

7. Schubert C, Naf J, Petukhov L, Laganenka L, Cherrak Y, Hardt WD. Strain-specific galactose utilization by commensal E. coli mitigates Salmonella establishment in the gut. PLoS Pathog. 2025;21(6):e1013232. Epub 20250627. doi: 10.1371/journal.ppat.1013232. PubMed PMID: 40577327.

8. Santamaria de Souza N, Cherrak Y, Andersen TB, Vetsch M, Barthel M, Kroon S, et al. Context-dependent change in the fitness effect of (in)organic phosphate antiporter glpT during Salmonella Typhimurium infection. Nat Commun. 2025;16(1):1912. Epub 2025/02/25. doi: 10.1038/s41467-025-56851-5. PubMed PMID: 39994176; PubMed Central PMCID: PMCPMC11850910.

9. Fuchs L, Dieterich CL, Melgarejo Ros E, Keller P, Sintsova A, Laganenka L, et al. The Gfr Uptake System Provides a Context‐Dependent Fitness Advantage to Salmonella Typhimurium SL 1344 During the Initial Gut Colonization Phase. Molecular Microbiology. 2025.

10. Daniel BBJ, Steiger Y, Sintsova A, Field CM, Nguyen BD, Schubert C, et al. Assessing microbiome population dynamics using wild-type isogenic standardized hybrid (WISH)-tags. Nat Microbiol. 2024;9(4):1103-16. Epub 2024/03/20. doi: 10.1038/s41564-024-01634-9. PubMed PMID: 38503975; PubMed Central PMCID: PMCPMC10994841.

11. Stecher Br, Hapfelmeier S, Müller C, Kremer M, Stallmach T, Hardt W-D. Flagella and chemotaxis are required for efficient induction of Salmonella enterica serovar Typhimurium colitis in streptomycin-pretreated mice. Infection and immunity. 2004;72(7):4138-50.

12. Schubert C, Hoos M, Sichert A, Näpflin N, Kroon S, Pulli S, et al. D-gluconate drives Salmonella growth during acute and chronic infection. bioRxiv. 2026:2026.04.07.716905. doi: 10.64898/2026.04.07.716905.

13. Cain AK, Barquist L, Goodman AL, Paulsen IT, Parkhill J, van Opijnen T. A decade of advances in transposon-insertion sequencing. Nat Rev Genet. 2020;21(9):526-40. Epub 2020/06/14. doi: 10.1038/s41576-020-0244-x. PubMed PMID: 32533119; PubMed Central PMCID: PMCPMC7291929.

14. Maier L, Diard M, Sellin ME, Chouffane ES, Trautwein-Weidner K, Periaswamy B, et al. Granulocytes impose a tight bottleneck upon the gut luminal pathogen population during Salmonella typhimurium colitis. PLoS Pathog. 2014;10(12):e1004557. Epub 2014/12/19. doi: 10.1371/journal.ppat.1004557. PubMed PMID: 25522364; PubMed Central PMCID: PMCPMC4270771.

15. Nishimura K, Johansen SK, Inaoka T, Hosaka T, Tokuyama S, Tahara Y, et al. Identification of the RsmG methyltransferase target as 16S rRNA nucleotide G527 and characterization of Bacillus subtilis rsmG mutants. Journal of bacteriology. 2007;189(16):6068-73.

16. Okamoto S, Tamaru A, Nakajima C, Nishimura K, Tanaka Y, Tokuyama S, et al. Loss of a conserved 7‐methylguanosine modification in 16S rRNA confers low‐level streptomycin resistance in bacteria. Molecular microbiology. 2007;63(4):1096-106.

17. Forbes JR, Gros P. Divalent-metal transport by NRAMP proteins at the interface of host–pathogen interactions. Trends in microbiology. 2001;9(8):397-403.

Attachment Submitted filename: response to reviewers 12082026.pdf

# Peer review: Decision Letter 1

**DOI:** 10.1371/journal.pone.0349327.r003

**Authors:** Mohammad Faezi Ghasemi

7 Sep 2026

WISH-barcoding of Salmonella Typhimurium ATCC14028s strains for population dynamics studies in vivo

PONE-D-26-20998R1

Dear Dr. Hardt,

We’re pleased to inform you that your manuscript has been judged scientifically suitable for publication and will be formally accepted for publication once it meets all outstanding technical requirements.

Within one week, you’ll receive an e-mail detailing the required amendments. When these have been addressed, you’ll receive a formal acceptance letter and your manuscript will be scheduled for publication.

An invoice will be generated when your article is formally accepted. Please note, if your institution has a publishing partnership with PLOS and your article meets the relevant criteria, all or part of your publication costs will be covered. Please make sure your user information is up-to-date by logging into Editorial Manager at Editorial Manager® and clicking the ‘Update My Information' link at the top of the page. For questions related to billing, please contact billing support.

If your institution or institutions have a press office, please notify them about your upcoming paper to help maximize its impact. If they’ll be preparing press materials, please inform our press team as soon as possible -- no later than 48 hours after receiving the formal acceptance. Your manuscript will remain under strict press embargo until 2 pm Eastern Time on the date of publication. For more information, please contact onepress@plos.org.

Kind regards,

Mohammad Faezi Ghasemi, Ph.D

Academic Editor

PLOS One

Additional Editor Comments (optional):

Reviewers' comments:

Reviewer's Responses to Questions

**Comments to the Author**

Reviewer #1: All comments have been addressed

Reviewer #2: All comments have been addressed

**********

2. Is the manuscript technically sound, and do the data support the conclusions?

Reviewer #1: Yes

Reviewer #2: Yes

**********

3. Has the statistical analysis been performed appropriately and rigorously?

Reviewer #1: Yes

Reviewer #2: Yes

**********

4. Have the authors made all data underlying the findings in their manuscript fully available?

The PLOS Data policy

Reviewer #1: Yes

Reviewer #2: Yes

**********

5. Is the manuscript presented in an intelligible fashion and written in standard English?

Reviewer #1: Yes

Reviewer #2: Yes

**********

Reviewer #1: eviewer Report for PLOS ONE

Manuscript Number: PONE-D-26-20998R1

Title: WISH-barcoding of Salmonella Typhimurium ATCC14028s for population dynamics studies in vivo

Authors: Christopher Schubert, et al.

General Evaluation

The authors have submitted a revised version of their manuscript detailing the adaptation and challenges of WISH-barcoding for Salmonella Typhimurium ATCC14028s. In this revision, the authors have made substantial efforts to address the concerns raised by the editor and reviewers during the first round of review. The addition of necessary clarifications regarding the rationale for using the ΔinvG and ΔssaV mutants, the inclusion of Supplementary Figure 1 detailing the Shannon Evenness Index (SEI) of the inocula, and the expanded discussion on the mechanistic basis of rsmG selection significantly strengthen the manuscript. The authors have also thoroughly revised the statistical methods section to clarify the tests used, notably explicitly stating that no multiple comparison corrections were applied.

The responses provided by the authors are comprehensive and adequately address the major scientific and methodological queries. The modifications enhance the clarity, rigor, and contextual depth of the study.

Detailed Assessment of Revisions

1. Clarity of the ΔinvG and ΔssaV Backgrounds (Editor Concern 1 & Reviewer 1 Concern 3):

The authors have successfully expanded the introduction to clearly define the roles of InvG (T3SS-1, essential for host cell invasion) and SsaV (T3SS-2, essential for intracellular survival and systemic dissemination). By explaining that these mutants were chosen to evaluate whether defects in epithelial invasion or systemic survival alter population stability during gut-luminal colonization, the rationale for their inclusion is now well-established and informative. Furthermore, the authors have addressed Reviewer 1's concern by adding data (Supplementary Figure 2, Supplementary Data 4 and 5) demonstrating that both mutant pools maintained the P3 plasmid and did not acquire rsmG SNPs during construction, which serves as a robust control.

2. Mechanism Underlying Background-Specific Effects and rsmG Selection (Editor Concern 2 & Reviewer 1 Concern 1a):

The authors have appropriately expanded the discussion regarding the background-specific effects and the mechanism of rsmG selection. They convincingly argue against growth-rate-dependent differences by noting comparable growth rates in LB among the wild-type, ΔinvG, and ΔssaV strains during routine inoculum preparation. Additionally, they clarify that RsmG is a 16S rRNA methyltransferase modifying G527. The loss of this methylation directly reduces streptomycin binding affinity, providing a strong, direct survival advantage under streptomycin selection during strain construction, independent of general colonization fitness. The authors properly bound the scope of the study by acknowledging that deciphering the precise downstream effects of RsmG loss on translational fidelity is beyond the current work.

3. Statistical Analysis Details (Editor Concern 3 & Reviewer 1 Concern 5):

The Statistical Analysis section has been significantly improved. The authors have explicitly stated that the two-tailed Mann-Whitney U test was used for independent groups and the Wilcoxon signed-rank test for paired comparisons within the same animal. Crucially, they have clarified that no correction for multiple comparisons was applied and that each comparison was treated as an independent hypothesis test, noting this as a limitation where relevant. This transparency is essential for rigorous reporting and satisfies the concerns raised.

4. Contextualization of WISH-barcoding vs. RB-TnSeq (Editor Concern 4):

The addition to the introduction adequately explains the advantage of WISH-barcoding for hypothesis-driven studies. By explicitly stating that RB-TnSeq relies on large numbers of mutant strains (up to 2000), making it susceptible to pronounced bottlenecks imposed by immune responses, the authors clearly differentiate the utility of the WISH-barcoding approach, which relies on a smaller number of strains.

5. Mouse Model Differences (Reviewer 1 Concern 2):

The authors have added a concise and accurate explanation regarding the genetic differences between the C57BL/6J and 129S6/SvEvTac mouse models. They highlight the functional status of Nramp1 (Slc11a1), a phagosomal-membrane transporter that restricts divalent metals (Fe$^{2+}$ and Mn$^{2+}$) to intracellular Salmonella, noting that its functionality in 129S6/SvEvTac confers better control of systemic infection. This provides necessary context for interpreting the SEI data and bottleneck severity across the two models.

6. Barcode-Specific Fitness Effects (Reviewer 2 Concern 1):

The authors addressed the possibility of specific barcodes (e.g., W115) conferring a fitness advantage by adding a statement to the discussion. They note that the same WISH barcodes have been used extensively in previous studies with S. Typhimurium SL1344 without any observed barcode-specific fitness effects, thereby making it unlikely that barcode identity influences strain fitness in their system. This is a reasonable and sufficient justification.

7. Data Accessibility (Editor Concern 5 & Reviewer 1 Concern 4):

The authors have committed to making all sequencing data fully accessible prior to publication under the specified ENA accession number (PRJEB81516). Furthermore, they have added a clear statement explaining that organ samples were excluded from WISH barcode assessment because traversal from the gut lumen to systemic sites imposes a population bottleneck resulting in a loss of barcode diversity.

8. Formatting and Minor Issues (Editor Concerns 6-13, Reviewer 1 Concerns 6-7):

The authors appear to have addressed the minor formatting issues, including adding details to the abstract regarding broader implications, clarifying the MIC assay biological replicates, and providing a comprehensive contents list for the Supplementary Data. The addition of the inoculum SEI data in Supplementary Figure 1 is particularly helpful in demonstrating that the instability arose during strain construction.

Conclusion

The authors have engaged constructively with the reviewers' comments and have made the necessary revisions to improve the manuscript. The revised manuscript is scientifically sound, the methodology is clear, and the conclusions are well-supported by the data. The study provides important insights into the methodological challenges associated with barcoding ATCC14028s and the unintended selection pressures during in vitro construction.

Recommendation: Accept in current form.

Reviewer #2: The authors have addressed comments from both reviewers. I endorse this manuscript for publication in this journal.

**********

what does this mean?). If published, this will include your full peer review and any attached files.

If you choose “no”, your identity will remain anonymous but your review may still be made public.

**Do you want your identity to be public for this peer review?**  For information about this choice, including consent withdrawal, please see our Privacy Policy

Reviewer #1: No

Reviewer #2: No

**********

# Editor report: Acceptance letter

**DOI:** 10.1371/journal.pone.0349327.r004

**Authors:** Mohammad Faezi Ghasemi

PONE-D-26-20998R1

PLOS One

Dear Dr. Hardt,

I'm pleased to inform you that your manuscript has been deemed suitable for publication in PLOS One. Congratulations! Your manuscript is now being handed over to our production team.

At this stage, our production department will prepare your paper for publication. This includes ensuring the following:

* All references, tables, and figures are properly cited

* All relevant supporting information is included in the manuscript submission,

* There are no issues that prevent the paper from being properly typeset

You will receive further instructions from the production team, including instructions on how to review your proof when it is ready. Please keep in mind that we are working through a large volume of accepted articles, so please give us a few days to review your paper and let you know the next and final steps.

Lastly, if your institution or institutions have a press office, please let them know about your upcoming paper now to help maximize its impact. If they'll be preparing press materials, please inform our press team within the next 48 hours. Your manuscript will remain under strict press embargo until 2 pm Eastern Time on the date of publication. For more information, please contact onepress@plos.org.

You will receive an invoice from PLOS for your publication fee after your manuscript has reached the completed accept phase. If you receive an email requesting payment before acceptance or for any other service, this may be a phishing scheme. Learn how to identify phishing emails and protect your accounts at https://explore.plos.org/phishing.

If we can help with anything else, please email us at customercare@plos.org.

Thank you for submitting your work to PLOS One and supporting open access.

Kind regards,

PLOS One Editorial Office Staff

on behalf of

Dr. Mohammad Faezi Ghasemi

Academic Editor

PLOS One
